# Dual‐Pronged Attack: pH‐Driven Membrane‐Anchored NIR Dual‐Type Nano‐Photosensitizer Excites Immunogenic Pyroptosis and Sequester Immune Checkpoint for Enhanced Prostate Cancer Photo‐Immunotherapy

**DOI:** 10.1002/advs.202302422

**Published:** 2023-08-06

**Authors:** He Wang, Zhangxin He, Yijian Gao, Dexiang Feng, Xuedong Wei, Yuhua Huang, Jianquan Hou, Shengliang Li, Weijie Zhang

**Affiliations:** ^1^ Department of Urology The First Affiliated Hospital of Soochow University Suzhou 215006 China; ^2^ Department of Urology Dushu Lake Hospital Affiliated to Soochow University Medical Center of Soochow University Suzhou Dushu Lake Hospital Suzhou 215000 China; ^3^ College of Pharmaceutical Sciences Soochow University Suzhou 215000 China

**Keywords:** immunotherapy, membrane anchoring, prostate cancer, pyroptosis, type I photosensitizer

## Abstract

Prostate cancer (PCa) is a frustrating immunogenic “cold” tumor and generally receives unsatisfied immunotherapy outcomes in the clinic. Pyroptosis is an excellent immunogenic cell death form that can effectively activate the antitumor immune response, promote cytotoxic T‐lymphocyte infiltration, and convert tumors from “cold” to “hot.” However, the in vivo application of pyroptosis drugs is seriously limited, and the upregulation of tumor PD‐L1 caused by photo‐immunotherapy further promotes immune escape. Herein, a new nano‐photosensitizer (YBS‐BMS NPs‐RKC) with pH‐response integrating immunogenic pyroptosis induction and immune checkpoint blockade is developed. The pH‐responsive polymer equipped with the cell membrane anchoring peptide RKC is used as the carrier and further encapsulated with the near‐infrared‐activated semiconductor polymer photosensitizer YBS and a PD‐1/PD‐L1 complex small molecule inhibitor BMS‐202. The pH‐driven membrane‐anchoring and pyroptosis activation of YBS‐BMS NPs‐RKC is clearly demonstrated. In vitro and in vivo studies have shown that this dual‐pronged therapy stimulates a powerful antitumor immune response to suppress primary tumor progression and evokes long‐term immune memory to inhibit tumor relapse and metastasis. This work provides an effective self‐synergistic platform for PCa immunotherapy and a new idea for developing more biocompatible photo‐controlled pyroptosis inducers.

## Graphical abstract

In this work, a pH‐driven membrane‐anchored near‐infrared dual type (type I and II) nano‐photosensitizer, YBS‐BMS NPs‐RKC, is developed for prostate cancer dual‐pronged immunotherapy. In vitro and in vivo studies have shown that this dual‐pronged therapy stimulates a powerful antitumor immune response to suppress primary tumor progression and evokes long‐term immune memory to inhibit tumor relapse and metastasis.

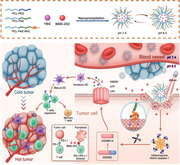

## 1 Introduction

Prostate cancer (PCa) is the second most common cancer and the fifth leading cause of cancer‐related death in men, with incidence rates increasing yearly.^[^ ^1^ ^]^ Early localized PCa patients can be treated with surgery or radiation therapy but may be accompanied by multiple complications such as urinary incontinence, sexual dysfunction, or bladder irritation.^[^ ^2^ ^]^ While patients with advanced PCa will overwhelmingly progress to castration‐resistant PCa after receiving endocrine therapy and chemotherapy. At this point, disease progression is accelerated, and survival is shortened.^[^ ^3^ ^]^ Thus, using a minimally invasive approach to completely remove tumor tissue at an early stage to protect against relapse and metastasis is an effective way to improve the prognosis of PCa patients.

As one of the focal therapies, photodynamic therapy (PDT) directly kills tumor cells by generating reactive oxygen species (ROS) at the tumor site, with selectivity, activatability, low toxicity, and reproducibility characteristics.^[^ ^4^ ^]^ Compared to ultraviolet/visible light, near‐infrared (NIR) light can penetrate deeper into the tissue with less tissue absorption and scattering, making them more suitable for clinical conduct.^[^ ^5^ ^]^ With the introduction of the vascular‐targeted photodynamic therapy (VTP) drug Tookad, the potential of PDT to treat PCa has been reconfirmed.^[^ ^6^ ^]^ Numerous studies have indicated that PDT for PCa can effectively remove tumor lesions, elicit antitumor immune effect, and improve the prognosis.^[^ ^7^ ^]^ Furthermore, the advent of nanobiotechnology has effectively improved the biocompatibility of photosensitizers, and the prostate's unique anatomical and physiological characteristics (near the natural lumen and slower local blood flow) make it more suitable for PDT using nano‐photosensitizers.^[^ ^8^ ^]^ However, there are still some restrictions of current nano‐photosensitizers in tumor PDT. On the one hand, the internal hypoxic environment of the tumor severely deprives the cytotoxicity of most photosensitizers; on the other hand, the apoptotic tumor cells induced with PDT are often phagocytosed by macrophages to undergo immunosilencing, which vastly reduces immunogenicity.^[^ ^9^ ^]^

Unlike most type II photosensitizers which strongly depend on surrounding O_2_, type I photosensitizers transfer electrons to surrounding substrates upon excitation and generate free radicals such as superoxide (∙O^2−^) and hydroxyl radical (HO∙), which are more suitable for tumor therapy.^[^ ^10^ ^]^ In particular, ∙O^2−^ can be further reacted to generate HO∙ with extremely high chemical reactivity under the action of superoxide dismutase and ferrous ions (Disproportionation reaction and Fenton reaction).^[^ ^11^ ^]^ HO∙ is able to react directly with various essential biomolecules to amplify the PDT effect.^[^ ^12^ ^]^ Notably, the cascade reaction process is associated with O_2_ production, which helps to alleviate deep tumor hypoxia and further improves the overall PDT efficiency.^[^ ^13^ ^]^ Pyroptosis is a highly efficient ICD form characterized by cell expansion, cell membrane perforation, and the release of massive cell contents.^[^ ^14^ ^]^ A large number of tumor antigens, damage‐related molecular patterns (DAMPs), and inflammatory cytokines released during immunogenic pyroptosis can effectively stimulate dendritic cells (DCs) maturation, activate tumor antigen‐specific T cells, promote cytotoxic T lymphocytes (CTLs) for tumor infiltration, convert immunogenic “cold” tumor into “hot” tumor, increase the response rate to immune checkpoint blockade (ICB) therapy and ultimately enhance the antitumor immune effect of the body.^[^ ^15^ ^]^ Recently, studies have shown that improving the cell membrane targeting of photosensitizers can convert low immunogenic apoptosis to high immunogenic pyroptosis triggered after PDT, providing a novel idea for the PIT.^[^ ^16^ ^]^ However, several reported cell membrane‐targeting photosensitizers are in small molecule form and primarily type II photosensitizers, which have significant limitations for in vivo therapeutic applications and deep tumor treatment.

**Scheme 1:**
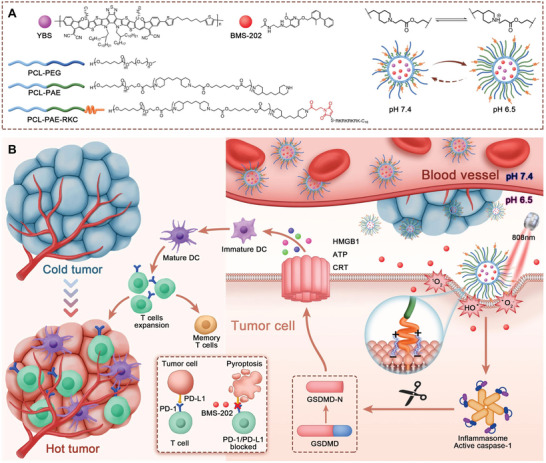
Schematic diagram of the composition, performance, and induction of prostate cancer self‐synergistic immunotherapy with YBS‐BMS NPs‐RKC.

In this study, we fabricated a pH‐driven membrane‐anchored NIR dual type (type I and II) nano‐photosensitizer, YBS‐BMS NPs‐RKC. By co‐encapsulating the semiconducting polymeric (SP) photosensitizer YBS and the PD‐1/PD‐L1 complex small molecule inhibitor BMS‐202 to achieve dual‐pronged photo‐immunotherapy (PIT) with immunogenic pyroptosis and ICB (**Scheme** **1**). To achieve pH‐driven cell membrane anchoring, the ligand‐switchable polymer hybrid micelle (LSPM) and the cell membrane anchoring peptide RKC (C‐RKRKRKRK‐C_16_) were used to construct the pH‐sensitive carrier for the nano‐photosensitizer. In particular, LSPM consists of the amphiphilic polymer poly(e‐caprolactone)‐*b*‐poly(ethylene glycol) (PCL‐*b*‐PEG) and the pH‐responsive polymer poly(e‐caprolactone)‐*b*‐poly(b‐amino ester) (PCL‐*b*‐PAE).^[^ ^17^ ^]^ The RKC peptide consists of the positively charged amino acid sequence RKRKRKRK and the palmitic acid fragment C_16._ Importantly, the RKC peptides are coupled to the PAE's end, achieving tumor site‐specific exposure. Briefly, hydrophobic and compacted PAE enables effective hiding of RKC peptide by the PEG in the circulation, while in the acidic conditions of the tumor microenvironment (TME, pH < 6.5), the protonation of PAE causes a hydrophilic transition, which in turn promotes the localized exposure of RKC peptide. On the tumor cell membrane, the RKRKRKRK unit enhances the membrane affinity of the nano‐photosensitizer through electrostatic interactions, and the C_16_ unit inserts into the cell membrane to achieve prolonged cell membrane anchoring of the nano‐photosensitizer.^[^ ^18^ ^]^ Under NIR photo‐irradiation, dual‐type photosensitizer YBS generated HO∙, ∙O^2−^, and ^1^O_2_ on the tumor cell membrane, effectively triggered immunogenic pyroptosis, increased intratumor CD8^+^ T cell infiltration and cytotoxic cytokine secretion, and initiated antitumor immunity. Moreover, the immune escape mediated by upregulation of PD‐L1 expression in tumor cells due to increased IFN‐γ secretion after the PIT could be effectively blocked by BMS‐202, further amplifying the effect of PIT.^[^ ^19^ ^]^ Thanks to the effective synergy of immunogenic pyroptosis induction and PD‐1/PD‐L1 blockade, YBS‐BMS NPs‐RKC exhibited potent tumor cell killing and antitumor immune activation abilities in in vitro and in vivo experiments, effectively inhibiting tumor growth in PCa subcutaneous tumor‐bearing mice model. Moreover, the long‐term immunological memory induced by PIT effectively prevented PCa relapse and metastasis. Collectively, this pH‐driven cell membrane‐anchored NIR dual‐type nano‐photosensitizer provides a new strategy for developing potent antitumor immunotherapeutic reagents.

## 2 Results and Discussion

### 2.1 Design, Synthesis, and Characterization of YBS NPs‐RKC

**Figure 1:**
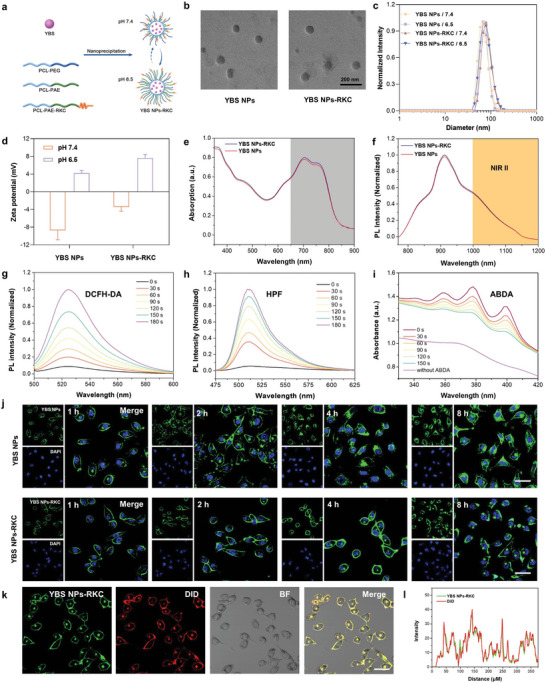
a) Schematic diagram of the preparation of nano‐photosensitizer YBS NPs‐RKC. b–d) TEM images, hydrodynamic diameters, and zeta potentials of YBS NPs and YBS NPs‐RKC at different pH solutions. e,f) Absorption and fluorescence spectrum of YBS NPs and YBS NPs‐RKC in PBS solution. g) Detection of total ROS generated by YBS NPs‐RKC (15 µg mL^−1^) using DCFH‐DA sensor (0.3 W cm^−2^ at 808 nm). h) Detection of HO∙ generated by YBS NPs‐RKC (15 µg mL^−1^) using HPF sensor (0.3 W cm^−2^ at 808 nm). i) Detection of ^1^O_2_ generated by YBS NPs‐RKC (15 µg mL^−1^) using ABDA sensor (0.3 W cm^−2^ at 808 nm). j) CLSM images of RM‐1 cells treated with YBS NPs or YBS NPs‐RKC at pH 6.5 at different time points, respectively. Blue fluorescence: DAPI; green fluorescence: the signals of YBS NPs or YBS NPs‐RKC, respectively. Scale bar: 10 µm. k,l) Co‐localization analysis of YBS NPs‐RKC with commercial cell membrane tracker DiD on RM‐1 cells. Green fluorescence: the signals of YBS NPs‐RKC; red fluorescence: the signals of DiD.

As a proof of concept, a polymer with NIR absorption (YBS) was synthesized by Stille polymerization. The detailed synthetic route was added in Figure S1 (Supporting Information). For further physicochemical property demonstration, the absorption and emission spectra of YBS were measured in THF solvent (Figure S2, Supporting Information). YBS has a good NIR absorption peak at about 670 nm and a strong NIR emission (700–1150 nm), which can be used for in vivo NIR‐II bioimaging and cancer theranostics. Then, we prepared nano‐photosensitizers YBS NPs‐RKC using pH‐responsive biocompatible polymers (PCL‐*b*‐PEG, PCL‐*b*‐PAE, and PCL‐*b*‐PAE‐RKC) by nanoprecipitation (**Figure** **1**a). The RKC peptide was linked to the PAE by the “click reaction” of the sulfhydryl group with the maleimide (Figures S3–S5, Supporting Information). Furthermore, the control nano‐photosensitizer, YBS NPs without RKC peptide, was synthesized similarly.

The physical and optical properties of YBS NPs‐RKC were investigated. As depicted in Figure 1b, YBS NPs‐RKC and YBS‐NPs showed spherical morphology with uniform size distribution. The DLS assay results further revealed that there was only a minor difference in the particle size of nanoparticles under different pH environments, indicating that the transition after PAE protonation did not substantially affect the particle size of YBS NPs and YBS NPs‐RKC (Figure 1c). Notably, both YBS NPs‐RKC and YBS‐NPs exhibited negative surface potentials at pH 7.4 in the presence of external PEG, while at pH 6.5, PAE protonation caused a rapid conversion to positive charge, implying a decisive phase transition at different pH environments (Figure 1d). YBS NPs‐RKC and YBS‐NPs exhibited similar NIR absorption and emission spectrum with maximum absorption at ≈710 nm and maximum emission at ≈910 nm, indicating that the conjugation of RKC peptides had almost no effect on the optical properties of the nano‐photosensitizers (Figure 1e,f). Furthermore, the emission spectrum of the nano‐photosensitizers can be up to 1150 nm, suggesting that they have excellent NIR‐II window imaging capability. In addition, the absorption spectrum of the YBS NPs‐RKC solution was almost unchanged under continuous irradiation of 808 nm laser for 10 min, verifying its excellent photostability (Figure S6, Supporting Information).

Then, the photodynamic performance of YBS NPs‐RKC was investigated. The generation of ROS for YBS NPs‐RKC under NIR (808 nm) irradiation was detected using an overall ROS sensor (2,7‐dichlorodihydrofluorescein, DCFH‐DA). As shown in Figure 1g and Figure S7 (Supporting Information), YBS NPs‐RKC significantly induced the enhanced fluorescence intensity of DCF at 524 nm under laser irradiation, which increased 11.4‐fold after 180 s, confirming its outstanding ROS production ability. Next, the types of generated ROS were further assessed with HO∙ sensor (HPF), ∙O_2_^−^ sensor (DHR123) as fluorescent indicators, and ^1^O_2_ sensor (ABDA) as absorption indicator, respectively.^[^ ^20^ ^]^ HO∙ and ∙O_2_^−^ are the representative type of type I ROS.^[^ ^21^ ^]^ HPF and DHR123 exhibited progressive fluorescence enhancement after oxidation with HO∙ and ∙O_2_^−^, respectively, which were used to detect the generation of type I ROS.^[^ ^22^ ^]^ As shown in Figure 1h and Figures S8 and S9 (Supporting Information), the fluorescence intensity of HPF at 510 nm was enhanced 17.9‐fold, and the fluorescence intensity of DHR123 at 525 nm was also increased 10.5 fold after 180 s of laser irradiation. ABDA is one of the most commonly used sensors for detecting type II ROS production, which exhibits a gradual decrease in absorbance after oxidation by ^1^O_2_.^[^ ^23^ ^]^ As displayed in Figure 1i and Figure S10 (Supporting Information), the absorption of ABDA at 378 nm gradually decreased with the extension of laser irradiation time, and the absorption intensity decreased by 42% after 150 s. In addition, the temperature of YBS NPs‐RKC solution under laser irradiation showed almost no change, indicating that YBS NPs‐RKC does not have photothermal conversion ability at this laser power (Figure S11, Supporting Information). All experimental results demonstrated that YBS NPs‐RKC is a dual‐type nano‐photosensitizer with outstanding photodynamic properties, which can effectively break through tumor hypoxia limitation and improve therapeutic efficacy.

Finally, the pH‐driven membrane anchoring ability of YBS NPs‐RKC was studied using confocal laser scanning microscopy (CLSM). For observation in the visible range, we added an AIE luminogen TPRA (excitation: 488 nm; emission: 630 nm) during the construction of YBS NPs‐RKC and YBS NPs.^[^ ^14c^ ^]^ To clarify the pH‐driven membrane anchoring ability of YBS NPs‐RKC, we evaluated the distribution of YBS NPs‐RKC and YBS‐NPs within RM‐1 cells (PCa cell line) under different pH culture environments (7.4 or 6.5). As illustrated in Figure 1j and Figure S12 (Supporting Information), YBS NPs‐RKC, and YBS NPs presented a scattered distribution in RM‐1 cells at pH 7.4, indicating that both nano‐photosensitizers had little interaction with the cell membrane in the physiological pH environment. In contrast, when the pH of culture medium was lowered to 6.5, a distinct green fluorescence was visible on the cell membrane, indicating that the protonated nano‐photosensitizers stayed on the cell membrane with the aid of electrostatic interaction. More importantly, compared to YBS NPs that started to enter the cells after 2 h of incubation gradually, the overwhelming majority of YBS NPs‐RKC remained stably anchored to the cell membrane after 8 h of incubation, which was attributed to the strong membrane anchoring ability of RKC peptide. Afterward, the green fluorescence of YBS NPs‐RKC overlapped well with the red fluorescence of the commercial cell membrane tracker DiD, again confirming the robust cell membrane anchoring ability of YBS NPs‐RKC (Figure 1k,l). In addition, co‐incubation experiments of YBS NPs‐RKC with mouse normal embryonic fibroblast 3T3 cells in a physiological environment revealed that only a minimal amount of nanoparticles were internalized by the cells after an incubation period of up to 4 h, which was significantly lower than that of RM‐1 cells. This further demonstrates the unique targeting ability of YBS NPs‐RKC on tumor cells under pH‐driven conditions (Figure S13, Supporting Information).

### 2.2 YBS NPs‐RKC Induces Immunogenic Pyroptosis

**Figure 2:**
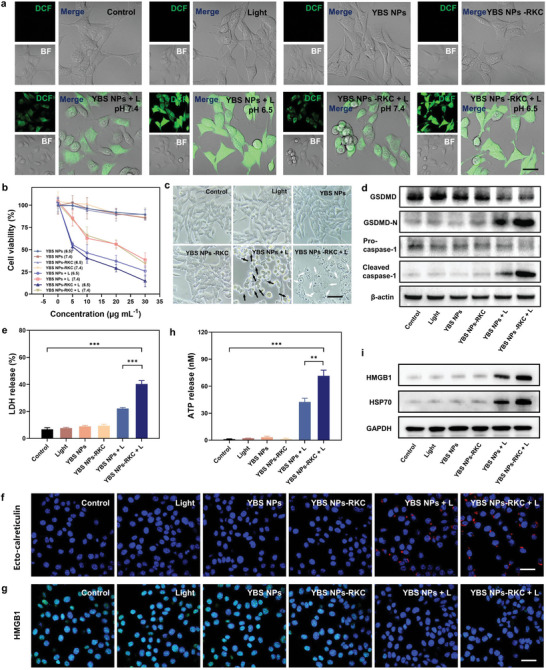
a) ROS production in RM‐1 cells was detected using DCFH‐DA sensor. [YBS] = 15 µg mL^−1^; 0.3 W cm^−2^ at 808 nm. Scale bar: 10 µm. b) Cell viabilities of RM‐1 cells treated with YBS NPs‐RKC or YBS NPs at different YBS concentrations or pH culture medium with or without NIR laser irradiation (*n* = 4). c) Representative images of RM‐1 cells after different treatments. White arrows (pyroptosis): cell swollen, expanded, large bubbles; black arrows (apoptosis): cell shrinking, solidification, apoptotic bodies. Scale bar: 20 µm. d) Western blot for cleaved Caspase‐1 and GSDMD‐N protein expressed after different treatments. e) Quantitative analysis of LDH release from RM‐1 cells with different treatments (*n* = 5). f,g) Representative confocal images showing the expression of ecto‐CRT or HMGB1 in RM‐1 cells with different treatments. Scale bar: 10 µm. h) Quantitative analysis of ATP release from RM‐1 cells with different treatments (*n* = 3). i) Western blot analysis of HMGB1 release and HSP70 release in the cell supernatants of RM‐1 cells with different treatments. Statistical analysis was performed by Student's *t*‐test (**P < 0.01, ***P < 0.001).

The ability to evoke immunogenic pyroptosis with YBS NPs‐RKC under laser irradiation was investigated in RM‐1 PCa cells. The intracellular ROS‐generating ability of YBS NPs‐RKC was measured using the fluorescence turn‐on probe DCFH‐DA. Under the oxidation of ROS, DCFH was converted to DCF to emit fluorescence. As shown in **Figure** **2**a, YBS NPs or YBS NPs‐RKC generated more ROS after laser irradiation under pH 6.5 incubation conditions, due to the positively charged nano‐photosensitizers after protonation, which could more easily enter the cells or anchor on the cell membrane. Accordingly, the protonated YBS NPs or YBS NPs‐RKC have a more substantial cell‐killing effect after NIR laser irradiation, consistent with the previously reported (Figure 2b).^[^ ^17^, ^24^ ^]^ Furthermore, attributing to the specific ability to generate type I ROS, YBS NPs‐RKC NPs exhibited sustained cytotoxicity even under hypoxic conditions (Figure S14, Supporting Information).

Notably, YBS NPs‐RKC exhibited a better cell‐killing effect at the same concentration than YBS NPs, which prompted us to explore more deeply the cell death patterns induced by YBS NPs‐RKC and YBS NPs after NIR laser exposure. Firstly, we observed and recorded the cell morphologies of each group under different treatments. As displayed in Figure 2c, YBS NPs‐treated cells displayed typical apoptotic features after NIR laser irradiation, i.e., smaller cell size, solidification, and the formation of apoptotic vesicles. In contrast, YBS NPs‐RKC‐treated cells exhibited typical features of cell pyroptosis, including swollen, expanded, and formed large bubble‐like protrusions after NIR laser irradiation.^[^ ^25^ ^]^ Furthermore, there were no significant cell morphological changes in the pure YBS NPs‐RKC‐treated group, the pure YBS NPs‐treated group, and the pure laser‐treated group, indicating the cell‐killing effects of YBS NPs‐RKC and YBS NPs were due to the ROS generated by them. Compared to the immune silencing that occurs with apoptosis, the large number of cellular contents released during cell pyroptosis can stimulate a more intense immunogenic activity, which enhances the body's antitumor immune response.^[^ ^9c,d^ ^]^ Western blot assay confirmed that YBS NPs‐RKC evoked cell pyroptosis in PCa RM‐1 cells after NIR laser irradiation by activating the classical caspase‐1/GSDMD pathway. As displayed in Figure 2d, YBS NPs‐RKC caused a significant increase in GSDMD‐N and cleaved‐Caspase‐1 expression in RM‐1 cells after NIR laser irradiation. ROS generated at the cell membrane site are recognized by the cell's pattern recognition receptors to promote the formation of downstream NLRP3 inflammasomes, activating Caspase‐1.^[^ ^26^ ^]^ Activated caspase‐1 cleaves the pyroptosis execution protein GSDMD to produce the GSDMD‐N terminus, which translocates into the cell membrane structure to form a plasma membrane pore, disrupting cellular integrity and causing the release of cellular contents.^[^ ^27^ ^]^ Moreover, YBS NPs‐RKC + L treatment evoked substantial lactate dehydrogenase (LDH) release from RM‐1 tumor cells, further demonstrating the robust cell pyroptosis‐inducing ability of this pH‐driven membrane‐anchored nano‐photosensitizer (Figure 2e).

Cell pyroptosis is a potent ICD form, and the DAMPs release level induced by YBS NPs‐RKC in RM‐1 tumor cells under NIR irradiation was further evaluated. As shown in Figure 2f, YBS NPs‐RKC+L treatment induces the translocation of intracellular calreticulin (CRT) and exposes them to the cell membrane surface, releasing “eat‐me” signals that promote DCs or their precursor cells to phagocytose dead or dying tumor cells for upregulation of antitumor immunity. Accordingly, the release levels of other DAMPs, such as HMGB1, HSP70, and ATP, were further investigated. The results showed that the HMGB1 levels in RM‐1 cells were significantly decreased after either YBS NPs + L or YBS NPs‐RKC + L treatment, while the concentrations of ATP, HMGB1 and HSP70 in the cell supernatant were all obviously increased (Figure 2g–i). Notably, the level of DAMPs release was more remarkable in YBS NPs‐RKC + L treatment compared with YBS NPs + L treatment, indicating that the cell pyroptosis evoked by YBS NPs‐RKC after NIR irradiation had better immunogenicity.

### 2.3 NIR‐II Imaging and In Vivo PIT

**Figure 3:**
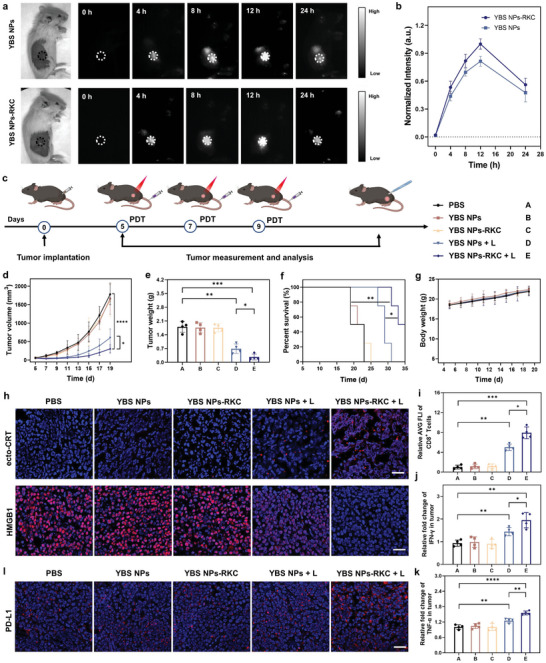
a) In vivo NIR‐II fluorescence imaging at different timepoints after YBS NPs or YBS NPs‐RKC administration (200 µL, [YBS] = 1 mg mL^−1^). b) Quantification of the image intensities in panel (a) (*n* = 3). c) Schedule of the treatment and subgroups of RM‐1 tumor‐bearing mice. d,e) RM‐1 tumor growth curves and end‐stage weights of each group (*n* = 4). f) Survival record of each group of mice. g) Body weight monitoring of tumor‐bearing mice under different treatments. h) Representative confocal images of ecto‐CRT and HMGB1 levels in RM‐1 tumor sections. Scale bar: 50 µm. i) Quantitative analysis of tumor‐infiltrating CD8^+^ T cells in RM‐1 tumors after different treatments. j,k) IFN‐γ and TNF‐α levels in the tumors of RM‐1‐tumor‐bearing mice. l) Representative confocal images of PD‐L1 expression levels in RM‐1 tumor sections. Scale bar: 50 µm. Statistical analysis was performed by Student's *t*‐test or log‐rank test (*P < 0.05, **P < 0.01, ***P < 0.001).

Photosensitizers with NIR‐II fluorescence imaging capability can significantly improve the spatial and temporal precision of PDT, contributing to the complete clearance of tumor lesions and the protection of normal tissue.^[^ ^28^ ^]^ YBS NPs‐RKC‐mediated NIR‐II imaging capability and PDT were further investigated in RM‐1 tumor‐bearing C57BL/6J mice. As depicted in **Figure** **3**a,b, the results of NIR‐II imaging studies at tumor enrichment time‐points revealed that YBS NPs‐RKC and YBS NPs had good tumor accumulation ability, and the fluorescence at the tumor site was consistently enhanced after intravenous injection and reached the highest intensity at 12 h. Compared to YBS NPs, YBS NPs‐RKC had higher fluorescence intensity at all time points, which may be attributed to the pH‐driven cell membrane anchoring ability to achieve more extended residence of tumor cells. Moreover, *ex vivo* organ imaging results indicated that YBS NPs‐RKC and YBS NPs were metabolized in vivo mainly through the liver, and the fluorescence signal of major organs started to weaken after 12 h of administration (Figure S15, Supporting Information).

Next, the in vivo PDT study was conducted based on the optimal time of enrichment of YBS NPs‐RKC and YBS NPs within the tumor as determined in the imaging study (Figure 3c). Mice were subcutaneously inoculated with RM‐1 cells, and this day was set as day 0. Mice were randomly divided into “PBS,” “YBS NPs,” “YBS NPs‐RKC,” “YBS NPs + L,” and “YBS NPs‐RKC + L” groups after the tumor volume grew to about 100 mm^3^ (day 5) and received the corresponding treatment (n = 4 mice per group). RM‐1 tumor‐bearing mice received NIR light‐irradiation (0.3 W cm^−2^ at 808 nm, 10 min) after 12 h of intravenous injection of YBS NPs‐RKC or YBS NPs (200 µL, [YBS] = 1 mg mL^−1^), which were repeated on days 7 and 9. Subsequently, the tumor growth and weight change of mice were continuously monitored. As shown in Figure 3d and Figure S16 (Supporting Information), tumor growth was not inhibited in either “YBS NPs‐RKC” or “YBS NPs” groups, and the mean tumor volumes on day 19 were ≈1607 or 1667 mm^3^, respectively, which were no statistically significant differences compared with the “PBS” group (≈1781 mm^3^). In contrast, laser irradiation significantly turned on the tumor suppressive ability of YBS NPs‐RKC and YBS NPs, which is consistent with the cytotoxicity study at the cellular level. Notably, compared with the “YBS NPs + L” group, the “YBS NPs‐RKC + L” group obtained better tumor growth inhibition, with the average tumor volume at day 19 only ≈304 mm^3^, which was 1.98‐ and 5.85‐fold smaller than that of “YBS NPs + L” and “PBS” groups, respectively. Similarly, the RM‐1 tumor weight data also demonstrated that “YBS NPs‐RKC + L” had the best antitumor effect, with the mean tumor weight of this group being only about one‐seventh of the mean tumor weight of the “PBS” group (Figure 3e). Furthermore, we also evaluated the tissue damage and proliferation inhibition of each group of tumor tissues by histological staining (Figure S17, Supporting Information). As observed by the hematoxylin‐eosin (H&E) staining, compared with the vigorous and tightly arranged tumor cells in the “PBS” group, extensive vacuoles or gaps appeared in the tumor tissues after YBS NPs + L or YBS NPs‐RKC + L treatment, the latter being more significant, indicating that a large number of tumor cells had died. The results of proliferating cell nuclear antigen (PCNA) staining also revealed that tumor proliferation was significantly inhibited after YBS NPs + L or YBS NPs‐RKC + L treatment. Moreover, a survival experiment was conducted to clarify that tumor cell pyroptosis based on YBS NPs‐RKC + L can provide a better therapeutic effect. As shown in Figure 3f, YBS NPs‐RKC + L treatment significantly prolonged the survival of mice, with 50% of treated mice surviving for more than one month. The data from the tumor suppression experiment and survival experiment indicated that the nano‐photosensitizer YBS NPs‐RKC with pH‐driven tumor cell membrane anchoring ability could obtain better treatment effects. In addition, there was no statistical difference in the body weight of mice between groups (Figure 3g), indicating that all treatments had good safety profiles.

To investigate the potential mechanism of efficient antitumor of YBS NPs‐RKC + L, we extracted tumor tissues from each group of mice after treatment, and initially explored the impact of PDT on the intratumor immune microenvironment of mice by immunofluorescence (IF) technique and enzyme‐linked immunosorbent assay (ELISA). Considering that ICD is the essential mediator in implementing PIT, we first analyzed the activation of DAMPs in each group of tumors. CLSM images indicated that DAMPs activation levels were significantly higher in tumor sections of the “YBS NPs + L” and “YBS NPs‐RKC + L” groups, as evidenced by substantial ecto‐CRT and HMGB1 release, and the latter more significantly (Figure 3h). CD 8^+^ T cells and their secreted cytokines (e.g., TNF‐α and IFN‐γ) exert the most pivotal and direct role in the antitumor immunity. As shown in Figure 3i–k and Figure S18 (Supporting Information), YBS NPs‐RKC + L therapy dramatically promoted CD8^+^ T cell infiltration and increased the secretion level of antitumor immune‐related cytokines within the tumor. These data further validated that YBS NPs‐RKC‐mediated immunogenic pyroptosis efficiently converts immunogenic “cold” tumor into “hot” tumor.

However, the antitumor function of CD8^+^ T cells was also impacted by the level of immune checkpoints (ICs) on the tumor cell surface. Representatively, high expression of PD‐L1 receptors on the tumor cell surface can bind to PD‐1 ligands on the T cell surface, promoting immune escape. As displayed in Figure 3l, PD‐L1 expression levels in tumor tissues were remarkably elevated after YBS NPs‐RKC‐mediated PDT, consistent with previous studies that a large amount of IFN‐γ secretion can promote tumor PD‐L1 expression.^[^ ^19^ ^]^

### 2.4 Dual‐Pronged PIT with YBS‐BMS NPs‐RKC

**Figure 4:**
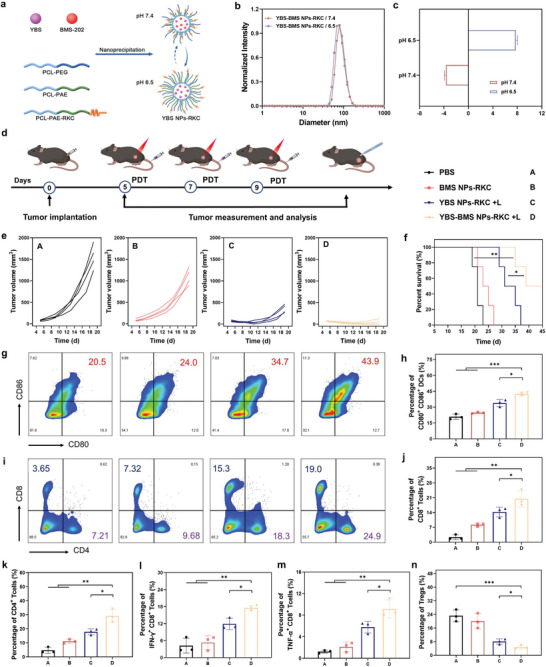
a) Schematic diagram of the preparation of nano‐photosensitizer YBS‐BMS NPs‐RKC. b,c) Hydrodynamic diameters and zeta potentials of YBS‐BMS NPs‐RKC at different pH solutions. d) Schedule of the treatment and subgroups of RM‐1 tumor‐bearing mice. e,f) RM‐1 tumor growth curves and survival record of each group of mice (*n* = 4). g) Representative cytometry patterns and h) quantification of matured DCs (CD11c^+^CD80^+^CD86^+^) in TDLNs (*n* = 3). i) Representative cytometry patterns and j,k) quantification of CD8^+^ T cells (CD3^+^ CD8^+^) and CD4^+^ T cells (CD3^+^ CD4^+^) in tumors (*n* = 3). l,m) Quantification of IFN‐γ^+^CD8^+^ T cells (CD3^+^ CD8^+^IFN‐γ^+^) and TNF‐α^+^CD8^+^ T cells (CD3^+^CD8^+^TNF‐α^+^) in tumors (*n* = 3). n) Quantification of Tregs (CD4^+^ FOXP3^+^) in tumors (*n* = 3). Statistical analysis was performed by Student's *t*‐test or log‐rank test (*P < 0.05, **P < 0.01, ***P < 0.001).

Blocking the binding between PD‐1 and PD‐L1 can effectively activate CD8^+^ T cells to strengthen the antitumor immune activation of the body. Therefore, BMS‐202, a potent small molecule inhibitor of the PD‐1/PD‐L1 complex, was introduced into the pH‐driven cell membrane‐anchored nano‐photosensitizer system (**Figure** **4**a). The encapsulation efficiency (EE%) and drug loading capacity (DLC%) of YBS and BMS‐202 were 78.22% and 10.68%, and 43.19% and 5.32%, respectively (Figure S19, Supporting Information). TEM images, hydrodynamic diameter, and surface potential test results showed that the introduction of BMS‐202 did not impact the particle size of the nano‐photosensitizer system and maintained its outstanding pH responsiveness (Figure 4b,c, and Figure S20, Supporting Information). Meanwhile, YBS‐BMS NPs‐RKC had good stability in PBS solution (Figure S21, Supporting Information). Next, the release ability of BMS‐202 was further investigated by in vitro simulation experiments. As shown in Figure S22 (Supporting Information), there was a moderate pH‐dependent release of BMS‐202, which could reach more than 60% at pH 6.5 for 48 h (simulated tumor microenvironment).

Subsequently, the tumor suppressive ability of YBS‐BMS NPs‐RKC turned on with NIR photo‐irradiation was evaluated in an RM‐1 subcutaneous tumor‐bearing mouse model. As illustrated in Figure 4d, mice were randomly divided into four groups named “PBS,” “BMS NPs‐RKC,” “YBS NPs‐RKC + L,” and “YBS‐BMS NPs‐RKC + L” group when the volume of tumor reached about 100 mm^3^, and each group was treated on day 5, day 7, and day 9, respectively. From the first treatment, tumor volumes and mice weights were recorded every 2 d. As shown in Figure 4e and Figure S23 (Supporting Information), the tumor growth curve data indicated that BMS NPs‐RKC treatment alone had little tumor suppressive effect, while the YBS NPs‐RKC + L treatment successfully suppressed tumor growth, but its efficacy was still unsatisfactory. In comparison, the immunogenic pyroptosis combined with ICB achieved by YBS‐BMS NPs‐RKC + L treatment had a surprising effect, with the mean tumor volume at day 19 being only ≈48 mm^3^, which was ≈7.4‐fold smaller than that of the YBS NPs‐RKC + L treatment group. Surprisingly, 50% of the mice in the “YBS‐BMS NPs‐RKC + L” group received complete tumor suppression and survived for more than 40 d (Figure 4f). The data on the tumor weights of mice in each group also suggested that YBS‐BMS NPs‐RKC + L treatment had a distinguished antitumor effect (Figure S24, Supporting Information). Furthermore, negligible changes in body weight and no damage to major organs were observed in all groups of mice during the 14‐d monitoring cycle, indicating that YBS‐BMS NPs‐RKC‐mediated PIT has high biosafety and biocompatibility (Figures S25 and S26, Supporting Information). Moreover, we also tested the routine blood and blood biochemical parameters of the mice. As shown in Figure S27 (Supporting Information), compared with untreated normal mice, the routine blood and blood biochemical parameters of mice administered with YBS‐BMS NPs‐RKC showed no pathological changes, further indicating that YBS‐BMS NPs‐RKC has good biosafety.

To further validate the potential mechanism of YBS‐BMS NPs‐RKC‐mediated PIT, tumor tissues and tumor‐draining lymph nodes (TDLNs) from treated mice were collected to evaluate the antitumor immune activation in each group of mice using flow cytometry (FC) and ELISA. As the most functional antigen‐presenting cell known, DCs are pioneering in initiating the body's antitumor immune response. They can efficiently uptake, process, and present antigens to activate the antitumor function of CTLs. As shown in Figure 4g,h, the levels of mature DCs in the lymph nodes of mice in the “YBS‐BMS NPs‐RKC + L” group were significantly higher, 2.0‐, 1.7‐, and 1.3‐fold higher than those in the “PBS,” “BMS NPs‐RKC,” and “YBS NPs‐RKC + L” groups, respectively. Correspondingly, the intra‐tumor CD8^+^ T cell infiltration level in the “YBS‐BMS NPs‐RKC + L” group was significantly higher than in other groups (Figure 4i,j). Similar outcomes were obtained for CD4^+^ T cell infiltration within the tumor (Figure 4k). Furthermore, to more deeply and directly reveal the strong antitumor immunity mediated by YBS‐BMS NPs‐RKC + L, we assayed the proportion of IFN‐γ^+^ CD8^+^ T cells and TNF‐α^+^ CD8^+^ T cells in tumor‐infiltrating CD8^+^ T cells, which are responsible for direct killing of tumor cells. As expected, YBS‐BMS NPs‐RKC‐mediated dual‐pronged PIT could better induce the secretion of cytotoxic cytokines by CD8^+^ T cells to evoke more robust antitumor immunity (Figure 4l,m and Figures S28 and S29, Supporting Information). Moreover, we also found that YBS‐BMS NPs‐RKC could effectively reduce the proportion of regulatory T cells (Tregs) within the tumor under NIR photo‐irradiation (Figure 4n and Figure S30, Supporting Information). Collectively, in vivo immune analysis results indicated that YBS‐BMS NPs‐RKC‐mediated PIT with NIR photo‐irradiation could effectively trigger immunogenic pyroptosis through pH‐driven cell membrane anchoring and block immune escape via BMS‐202 interfering with PD‐L1/PD‐1 interaction, thus generating more powerful antitumor immune response and obtaining better tumor suppression.

### 2.5 YBS‐BMS NPs‐RKC‐Mediated Long‐Term Immunological Memory In Vivo

**Figure 5:**
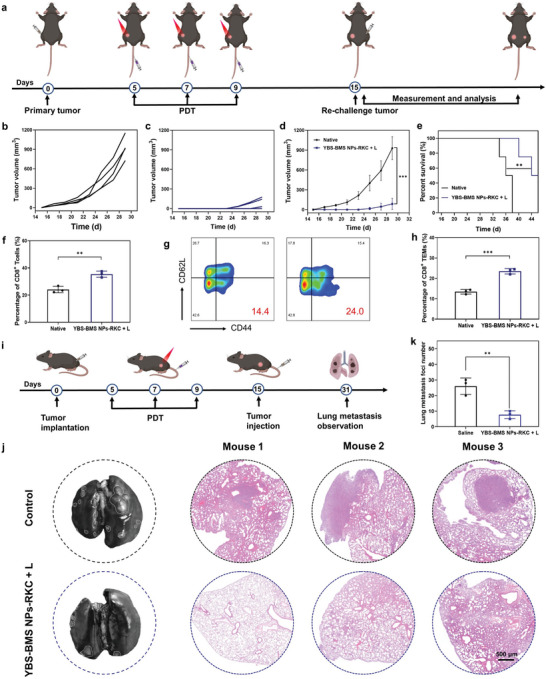
a) Schedule of the PCa recurrence experiment. b–d) Individual and average tumor growth records in re‐challenged RM‐1 tumors inoculated on native or cured mice (*n* = 4). e) Survival curves of native and cured mice after tumor re‐challenge. f) Quantification of CD8^+^ T cells (CD3^+^ CD8^+^) in spleens (*n* = 3). g) Representative cytometry patterns and h) quantification of effective memory CD8^+^ T cells (CD3^+^CD8^+^CD44^+^CD62^−^) in spleens (*n* = 3). i) Schedule of the PCa lung metastasis experiment. j) Representative images of the India ink‐stained whole lungs and H&E‐stained tissue sections. Scale bar = 500 µm. k) Number of lung tumor foci (*n* = 3). Statistical analysis was performed by Student's *t*‐test or log‐rank test (*P < 0.05, **P < 0.01, ***P < 0.001).

Relapse and metastasis of PCa are important causes of tumor‐related death.^[^ ^3^ ^]^ The establishment of immunological memory can provide long‐term protection to the organism and effectively protect against tumor relapse and metastasis.^[^ ^29^ ^]^ Therefore, the tumor re‐challenge model was used to assess whether immunological memory was effectively elicited and established in the body after YBS‐BMS NPs‐RKC + L treatment (**Figure** **5**a). Tumor‐bearing mice after PIT were re‐inoculated with RM‐1 tumor cells. Impressively, tumor growth records, end weights, and survival assay data indicated that YBS‐BMS NPs‐RKC‐mediated dual‐pronged PIT significantly suppressed tumor relapse and prolonged survival in RM‐1 tumor‐bearing mice. (Figure 5b–e and Figure S31, Supporting Information). In contrast, rapid tumor growth was observed in age‐ and weight‐matched normal mice. To identify the mechanism by which YBS‐BMS NPs‐RKC + L treatment suppressed RM‐1 PCa relapse, mice spleen tissues were extracted for immunological memory activation analysis. FC analysis results revealed that YBS‐BMS NPs‐RKC + L‐mediated dual‐pronged PIT effectively promoted the proportion of CD8^+^ T cells in the spleen by 1.5‐fold relative to native mice (Figure 5f and Figure S32, Supporting Information). More importantly, the proportion of effector memory T cells (TEMs) in the spleen of mice after YBS‐BMS NPs‐RKC + L treatment, a class that directly enters peripheral tissues to exert cytotoxic memory cells, was further evaluated. As shown in Figure 5g,h, the proportion of TEMs in the spleen of mice after dual‐pronged PIT was obviously higher than that in the native group. The results confirmed that YBS‐BMS NPs‐RKC could effectively stimulate tumor‐specific immunological memory responses with NIR photo‐irradiation in RM‐1 tumor‐bearing mice to protect against tumor relapse.

In clinical practice, lung metastasis is the more common type of distant metastasis among patients with PCa.^[^ ^30^ ^]^ Considering that YBS‐BMS NPs‐RKC‐mediated dual‐pronged PIT, as demonstrated in the above study, can effectively activate the organism's antitumor immunity and facilitate the maintenance of the body's immunological memory, we would like to evaluate whether it could exert an inhibitory effect in the process of PCa lung metastasis. According to the experiment schedule presented in Figure 5i, the PCa lung metastasis model was established. Whole‐lung ink‐stained tumor foci counts and H&E staining results revealed that tumors developed promptly in the lungs of native mice after intravenous injection of RM‐1 cells, while the YBS‐BMS NPs‐RKC + L‐treated group had only isolated foci, significantly fewer than the former (Figure 5j,k). This remarkable result reaffirmed that YBS‐BMS NPs‐RKC‐mediated dual‐pronged PIT could activate systemic antitumor immunological memory effects in PCa‐bearing mice and suggested its potential in personalized immunotherapy.

## 3 Conclusion

In summary, we developed a pH‐driven cell membrane‐anchored NIR dual‐type nano‐photosensitizer YBS‐BMS NPs‐RKC for dual‐pronged PIT of PCa. On the one hand, type I and type II ROS production by cell membrane localization under NIR laser irradiation can efficiently elicit immunogenic pyroptosis to convert immunogenic “cold” tumor into “hot” tumor; on the other hand, the timely release of BMS‐202 can effectively block the inevitable immune escape upregulation brought by the PIT process. It is proven that RKC peptides can be specifically exposed in the TME with the aid of pH‐responsive polymeric micelles LSPM to flexibly promote cell membrane anchoring of nano‐photosensitizer, resulting in cell membrane‐localized ROS production to activate caspase‐1/GSDMD pathway‐induced immunogenic pyroptosis. By synergizing immunogenic pyroptosis induction and ICB, this dual‐pronged PIT strategy can significantly promote CTLs infiltration in RM‐1 PCa, broadly stimulate a powerful antitumor immunity, effectively inhibit primary tumor growth, and establish long‐term immunological memory to protect against tumor relapse and metastasis. To the best of our knowledge, this is the first cell membrane‐anchored nano‐photosensitizer that induces immunogenic pyroptosis. The encapsulation of the nanocarrier significantly improves the biocompatibility of photosensitizer, and the pH‐responsive design maximally reduces nonspecific clearance in the blood as well as the generation of dual‐type ROS to break through the deep tumor hypoxia limitation effectively. Moreover, applying NIR windows allows for more precise and thorough imaging detection and treatment of tumor lesions. This strategy opens a platform for PIT of PCa and presents a new idea for the development and biological application of potent immunogenic pyroptosis inducers.

## Conflict of Interest

The authors declare no conflict of interest.

## Supporting information

Supporting Information

## Data Availability

The data that support the findings of this study are available from the corresponding author upon reasonable request.
