# Supplementary material for: Dual‐Pronged Attack: pH‐Driven Membrane‐Anchored NIR Dual‐Type Nano‐Photosensitizer Excites Immunogenic Pyroptosis and Sequester Immune Checkpoint for Enhanced Prostate Cancer Photo‐Immunotherapy
Source: Adv Sci (Weinh). 2023 Aug 6;10(28):2302422. doi: 10.1002/advs.202302422 (PMC10558672; doi:10.1002/advs.202302422)
Supplement: Supplementary file 1 — Supporting Information [file ADVS-10-2302422-s001.pdf]

ADVANCED SCIENCE
Open Access

Supporting Information

for *Adv. Sci.*, DOI 10.1002/advs.202302422

Dual-Pronged Attack: pH-Driven Membrane-Anchored NIR Dual-Type Nano-Photosensitizer Excites Immunogenic Pyroptosis and Sequester Immune Checkpoint for Enhanced Prostate Cancer Photo-Immunotherapy

He Wang, Zhangxin He, Yijian Gao, Dexiang Feng, Xuedong Wei, Yuhua Huang, Jianquan Hou, Shengliang Li* and Weijie Zhang**

Supporting Information

Dual-Pronged Attack: pH-Driven Membrane-Anchored NIR Dual-Type Nano-photosensitizer Excites Immunogenic Pyroptosis and Sequester Immune Checkpoint for Enhanced Prostate Cancer Photo-Immunotherapy

He Wang, Zhangxin He, Yijian Gao, Dexiang Feng, Xuedong Wei, Yuhua Huang, Jianquan Hou, Shengliang Li* and Weijie Zhang**

H. Wang, Prof. X. Wei, Prof. Y. Huang, Prof. J. Hou, and Dr. W. Zhang
Department of Urology, The First Affiliated Hospital of Soochow University, Suzhou 215006, China
E-mail: houjianquan@suda.edu.cn (J. Hou); zhangweijie@suda.edu.cn (W. Zhang)

Y. Gao, and Prof. S. Li
College of Pharmaceutical Sciences, Soochow University, Suzhou 215000, China.
E-mail: lishengliang@suda.edu.cn

Z. He, D. Feng, Prof. J. Hou, and Prof. W. Zhang
Department of Urology, Dushu Lake Hospital Affiliated to Soochow University, Medical Center of Soochow University, Suzhou Dushu Lake Hospital, Suzhou 215000, China

Experimental Section

Materials

All chemical reagents (unless otherwise specifically mentioned) were purchased from Shanghai Aladdin Biochemical Technology Co., Ltd. PCL-*b*-PEG, PCL-*b*-PAE, PCL-*b*-PAE-RKC were synthesized by Xi'an Ruixi Biotechnology Co., Ltd. Fetal bovine serum (FBS) and RMPI-1640 culture medium were purchased from Shanghai VivaCell Biosciences Ltd. Penicillin-streptomycin (PS) and 0.25% Trypsin-EDTA were purchased from Gibco. The ATP kits and LDH kits were purchased from Beyotime Biotechnology. Anti-Calreticulin antibody (ab92516), anti-HSP70 (ab2787), anti-HMGB1 antibody (ab18256), anti-Casapse-1 antibody (ab207802), anti-GSDMD antibody (ab215203), anti-GAPDH antibody (ab8245) and anti-β-actin antibody (ab8226) were purchased from Abcam Trading Co., Ltd. Rabbit IgG (H+L). The mouse RM-1 PCa cell line was purchased from Procell Life Science & Technology Co., Ltd.

Characterization

Bruker Avance 400 MHz spectrometer was used to record ^{1}H NMR. Transmission electron microscopy (TEM) images were obtained on Cryogenic TEM (Talos L120C G2, Czech). Dynamic light scattering (DLS) was carried out on a Malvern Nano-ZS90. Shimadzu UV-1800 UV-vis spectrophotometer was used to measure the UV–vis absorption spectrum. In Vivo NIR-II imaging system (Wuhan Guangyingmei, China) was used for small animal NIR-II fluorescence imaging. FCM Calibur (BD, USA) was used to perform flow cytometry. CLSM images were imaged on an LSM 800 (Zeiss, Germany).

Synthesis of YBS

Under N_2 atmosphere, Y6-OD-2Br (CAS: 2418532-43-9, 95 mg, 0.05 mmol) and 1,6-bis(5-(triMethylstannyl) thiophen-2-yl) hexane (29 mg, 0.05 mmol) were dissolved in anhydrous toluene (10 mL) in a 25 mL necked flask. N_2 injection was continued for 10 minutes, and Pd $(PPh_3)_4$ (12 mg, 0.01 mmol) was slowly added to the flask. The mixture was stirred at 110 °C for 24 h and cooled to room temperature, washed with water and chloroform. After that, anhydrous $MgSO_4$ was added to $CHCl_3$ to remove water, and then 40 mL of methanol was filtered and added to precipitate the crude product after concentration. Afterwards, the product was collected and further purified by Soxhlet extraction with CH_3OH, n-hexane, acetone and chloroform, following by recrystallization in methanol to obtain a blue-green solid. ^{1}H NMR (400 MHz, $CDCl_3$) δ 8.94 (s, 2H), 7.89 (s, 4H), 7.41 (s, 2H), 6.83 (s, 4H), 4.76 (s, 4H), 2.85 (s,

4H), 2.49 – 2.19 (m, 4H), 1.68 (s, 4H), 1.43 (s, 4H), 1.25 (s, 102H), 0.88 (s, 18H). GPC (THF, polystyrene standard), M_w: 1.09 × 10^4 g mol^{-1}, PDI: 1.10.

Preparation of Nano-photosensitizers

Preparation of YBS-BMS NPs-RKC: In short, 2 mg of PCL-b-PEG, 1.5 mg of PCL-b-PAE, and 0.5 mg of PCL-b-PAE-RKC were dissolved together with 1 mg of YBS and 1 mg of BMS-202 in 1 mL of THF. Then, drop the mixture into 9 mL of dd H_2O (PH = 3) at a uniform speed under an ultrasonic environment, and ultrasonic until it is thoroughly mixed. Next, dialysis was performed in dd H_2O (pH = 7.4) for 24 h using a dialysis bag (MWCO: 5000 Da), with fluid changes every 4 h. Finally, the product is obtained after filtration (0.22 μm) and centrifugation (4000 rpm, 7 min). YBS NPs, YBS NPs-RKC and BMS NPs-RKC were prepared in a similar way.

Encapsulation efficiency (EE) and Drug Loading Capacity (DLC)

The standard absorption curve was made according to the ultraviolet absorption of YBS or BMS-202 solution with different concentrations. Using the absorption curve as a standard, the concentration of YBS or BMS-202 was determined according to the absorption value. Afterward, the encapsulation efficiency (EE) and drug loading capacity (DLC) of YBS or BMS-202 were calculated using Eq. :

EE% = (mass of loaded drug)/(initial mass of drug) × 100%;

DLC% = (mass of loaded drug)/(mass of drug-loaded nanoparticles) × 100%.

BMS-202 Release

The in vitro release property of BMS-202 from YBS-BMS NPs-RKC was investigated via dialysis diffusion. In brief, 1 mg YBS-BMS NPs-RKC was placed in a dialysis bag with a molecular weight cut-off of 1000 Da and dialyzed with 30 mL PBS (pH 7.4, or 6.5) containing 0.1% Tween 80 accompanied by shaking (100 rpm) at 37 °C. A total of 2 mL of the solution was collected at the predetermined time, followed by supplement with the same volume to maintain a stable volume. Cumulative drug release was detected and calculated using UV–vis absorption spectrum. The percentage of drugs released was calculated using Eq. :

Drug released (%) = $(C_t \times V + Y) / M \times 100$

where V is the total volume; C_t is the concentration of drug at t time; Y is the total amount of drug collected before t time; and M is the original content for dialysis.

Transmission Electron Microscopy (TEM)

Add 10 μL of the YBS NPs, YBS NPs-RKC, or YBS-BMS NPs-RKC solutions slowly dropwise onto a clean copper grid, wait for the grid to dry, and then take pictures with TEM.

Determination of Particle Size and Zeta Potential

The YBS NPs, YBS NPs-RKC, or YBS-BMS NPs-RKC solutions with different pH (6.5 and 7.4) were prepared separately at a concentration of 20 μg/ml, and their particle size and potential were measured with Nano ZS90 (Zetasizer Malvern, USA).

ROS Detection

Total ROS detection: DCFH probe was used to detect the total ROS generation capacity of YBS NPs-RKC. The nano-photosensitizer stock solution was added to the DCFH aqueous solution (5 μM) to prepare a YBS NPs-RKC solution with a final concentration of 15 μg/mL. Under 808 nm laser irradiation (300 mW/cm^2), the total ROS generation capacity of the nano-photosensitizer was calculated by recording the fluorescence spectrum change.

Type I ROS detection ($HO\bullet/\bullet O_2^-$): HPF and DHR123 probes were used to detect the type I ROS generation capacity of YBS NPs-RKC. The nano-photosensitizer stock solution was added to HPF/DHR123 aqueous solution (10 μM) to prepare YBS NPs-RKC solution with a final concentration of 15 μg/mL. Under 808 nm laser irradiation (300 mW/cm^2), the type I ROS production capacity of the nano-photosensitizer was calculated by recording the change of fluorescence spectrum.

Type II ROS detection ($^1O_2$): ABDA probe was used to detect the type II ROS generation capacity of YBS NPs-RKC. The nano-photosensitizer storage solution was added to ABDA solution (50 μM) to prepare YBS NPs-RKC solution with a final concentration of 15 μg/mL. Under 808 nm laser irradiation (300 mW/cm^2), the type II ROS production capacity of the nano-photosensitizer was calculated by recording the change of UV absorption spectrum.

Cell Culture

Murine prostate cancer cell line RM-1 was cultured in complete RPMI 1640 culture medium including 10% FBS and 1% PS with an atmosphere of 5% CO_2 at 37 ^oC. We used 3-(N-Morpholino) propanesulfonic acid (MOPS) as a buffer to adjust the pH of the cell culture medium. MOPS is an amphoteric buffer that can effectively adjust the pH range of the medium from 6.5-7.9. Briefly, 2092.6 mg of MOPS was added to 500 mL of 1640 medium to prepare a

20 mM concentration of MOPS buffer. After complete dissolution, 1M dilute hydrochloric acid was added to adjust the pH. The pH was monitored using a pH detector during the adjustment.

Cell Membrane Targeting Study

RM-1 cells were pre-spread in confocal dishes and grown to 50-70% density before use. Cells were washed twice with 1×PBS buffer and incubated with fresh cell culture medium (pH 6.5 or 7.4) containing YBS NPs or YBS NPs-RKC ([YBS] = 15 μg/mL) for different times. Images of the cells were obtained on CLSM.

To co-stain with commercial membrane tracking agents, RM-1 cells were first treated with YBS NPs or YBS NPs-RKC (15 μg/ml) for 2 h. After washing twice with 1×PBS buffer, cells were treated with the membrane tracking agent DiD (2 μM) for 30 min. After 1×PBS washing, observation was performed.

ROS Detection in RM-1 Cells

RM-1 cells (4×10^4 /well) were pre-spread on the confocal chambers. The next day, the serum-free medium (pH = 7.4 or 6.5) containing YBS NPs or YBS NPs-RKC ([YBS] = 15 μg/mL) was changed to continue incubation for 8 h at 37 ^{o}C. Subsequently, cells were washed with 1×PBS solution and then incubated with DCF-DA (20 μM) solution for 30 min for staining. Finally, washed with PBS and imaged immediately after 808 nm photo-irradiation (300 mW/cm^2, 6 min). (excitation: 488 nm; emission: 500-550 nm).

Cytotoxicity Study

For the dark toxicity assay: RM-1 cells (3×10^3/well) were seeded in 96 well plates. After cell attachment, incubation with various concentrations of of YBS NPs-RKC or YBS NPs solution (pH = 6.5 or 7.4, 4 replicate wells per group). After 24 h, MTT was added and cultured for 4 h at 37 ^{o}C. Subsequently, the solution was discarded, 150 μL of DMSO was added to each well, dissolved and assayed using an enzyme marker.

For phototoxicity experiments: RM-1 cells (3×10^3/well) were seeded in 96 well plates. After cell attachment, incubation with various concentrations of of YBS NPs RKC or YBS NPs solution (pH = 6.5 or 7.4, 4 replicate wells per group). After 8 h of incubation, the solution was changed and irradiated with 808 nm laser (300 mW/cm^2) for 6 min. Continue incubation for 24 h and then add MTT and incubate for 4 h. Subsequently, the solution was discarded, 150 μL of DMSO was added to each well, dissolved and assayed using a microplate reader.

LDH Release Assay

The amount of LDH released from each group of cells was measured according to the LDH release assay kit, and the absorbance was measured at 490 nm. According to the instructions, RM-1 cells were divided into "blank group," "control group," "light group," "YBS NPs group," "YBS NPs +L group," "YBS NPs-RKC group," and "YBS NPs-RKC +L group" (5 replicate wells per group). After 8 h of incubation, the medium was changed, and the light was applied (300 mW/cm^2, 6 min). For measurements, the cell supernatant was extracted by centrifugation at 400 g for 5 min, and 120 μL of supernatant was added to the corresponding wells of a new 96 well plate. Then 60 μL of LDH detection reagent was added to each well, mixed, and incubated for 30 min in the dark. The absorbance (490 nm) was measured with a microplate reader.

ATP Release Assay

Chemiluminescence intensity was measured based on the ATP release from each group of cells by the ATP assay kit. According to the instructions, RM-1 cells were divided into "blank group," "control group," "light group," "YBS NPs group," "YBS NPs +L group," "YBS NPs-RKC group," and "YBS NPs-RKC +L group" (3 replicate wells per group). After 8 h of incubation, the medium was changed, and the light was applied (300 mW/cm^2, 6 min). For measurements, the cells were centrifuged at 1000 rpm for 3 min to obtain the cell cultural medium samples. The ATP assay working solution was prepared according to the instructions and added to an opaque 96-well black plate (100 μL/well). Finally, 20 μL of cell supernatant was added to each well after 5 min at room temperature, and the wells were mixed quickly before using microplate reader.

Immunofluorescence

RM-1 cells (4×10^4/well) were pre-spread on confocal chambers. After cell attachment, the cells were treated with various concentrations of of YBS NPs-RKC or YBS NPs solutions (pH = 6.5). After 8 h, the medium was changed and irradiated with 808 nm laser (300 mW/cm^2, 6 min), and incubation was continued for 6 h at 37 ^{o}C. Subsequently, cells were washed in PBS and then fixed with 4% PFA for 15 minutes. Next, cells were incubated overnight at 4 ^{o}C with primary antibodies (1:200) after washing with PBS. The next day, cells were further treated with fluorescent secondary antibodies (1:200) for 60 min at RT, nuclei were stained with DAPI, and then photographed under CLSM.

In Vivo Safety Assessment

The vital organs of each group of mice were taken, fixed, sectioned and stained with H&E, and the staining results were compared with the control mice to evaluate the in vivo safety of nano-photosensitizers.

In Vivo Time Point Imaging

The NIR-II fluorescence images were performed on RM-1 tumor-bearing mice. When the tumor volumes grew to about 100 mm^3, 200 μL of YBS NPs or YBS NPs-RKC solution was injected into the mice through the tail vein. Imaging was performed using the In Vivo NIR-II imaging system at 0, 4, 8, 12, and 24 h after injection, respectively.

In Vivo Tumor Inhibition Study

Tumors were established by subcutaneous injection of 2×10^6 RM-1 cells (RPMI-1640, 60 μL). According to the study schedule, the mice were randomly grouped when the tumor volume reached approximately 100 mm^3. The nano-photosensitizer solutions were administered intravenously on days 5, 7, and 9, respectively, and irradiated with NIR photo-irradiation (0.3 W/cm^2 at 808 nm 10 min) after 12 hours. At the same time, the tumor volume and weight changes of the mice were monitored continuously for 14 days.

$V = W^2 \times L/2$. (L: long diameter, W: short diameter).

Analysis of Immune Cells

For flow cytometric analysis, lymph nodes, tumors, and spleens of each group of mice were obtained to prepare single-cell suspensions for flow cytometric analysis. Briefly, the collected tissues were ground and passed through a 70 μm filter membrane to obtain a single cell suspension. Among them, spleen tissues need to be processed by erythrocyte lysis to remove red blood cells. Subsequently, the cells were dispersed in 96-well plates for staining with a stain volume of 100 ul and incubated at 4 ^oC for 30 min. Finally, the cells were centrifuged and resuspended using PBS and transferred to flow tubes for detection. For IFN-γ, TNF-α and FOXP3 staining, fixation and Permeabilization were required. Antibodies used in this study were listed in Table S1.

Immunofluorescence assays detected tumor ecto-CRT, HMGB1, and PD-L1 expression. ELISA kits (SinoBiological, China) were used to detect cytokines levels (TNF-α and IFN-γ) in serum.

Table S1. Antibodies used for flow cytometry in this study.

Antibodies	Company	Channel
Anti-mouse CD3	Biolegend	FITC
Anti-mouse CD4	Biolegend	PE-Cy7
Anti-mouse CD8	Biolegend	PE-594
Anti-mouse CD45	Biolegend	APC-fire750
Anti-mouse CD11c	Biolegend	BV421
Anti-mouse CD80	Biolegend	APC
Anti-mouse CD86	Biolegend	PE
Anti-mouse IFN-γ	Biolegend	APC
Anti-mouse TNF-α	Biolegend	PE
Anti-mouse FOXP3	Biolegend	PE
Anti-mouse CD44	Biolegend	APC
Anti-mouse CD62L	Biolegend	PE

In Vivo Tumor Relapse and Metastasis Experiment

For tumor relapse studies: primary subcutaneous tumors (2×10^6 RM-1 cells) were established in mice according to the experimental schedule, and recurrent tumors were established on the contralateral side of the primary tumor on day 15 after YBS-BMS NPs-RKC-mediated photoimmunotherapy. Subsequently, tumor growth and mouse weight changes were monitored compared to untreated native mice. Finally, the spleen tissues of mice were obtained for immune memory analysis at the end of the experiment.

For tumor metastasis studies: primary subcutaneous tumors (2×10^6 RM-1 cells) were established in mice according to the experimental schedule, and tumor cells (100 μL, 2×10^5) were injected intravenously on day 15 after YBS-BMS NPs-RKC-mediated photo-immunotherapy. Subsequently, mice were reasonably executed on day 31 and lungs were obtained for tumor nodule counting and H&E staining analysis.

Statistical Analysis:

GraphPad Prism 8.0 was employed for statistical analysis and plotting. Quantitative data were represented as mean ± SD. Statistical comparisons were analyzed by Student's t-test. Significance was set as $*p < 0.05$, $**p < 0.01$, *and* $***p < 0.001$. Survival curves were studied using a log-rank test and plotted using the Kaplan-Meier method.

Supplementary results

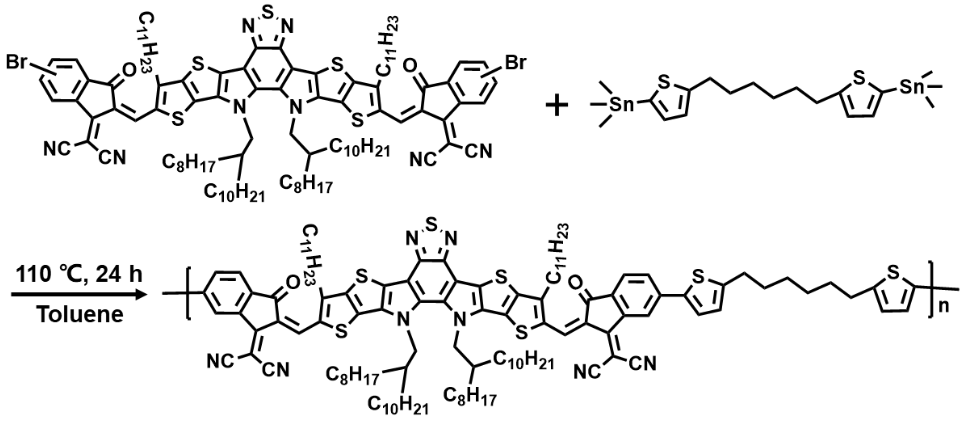

Figure S1. Synthetic route to YBS.

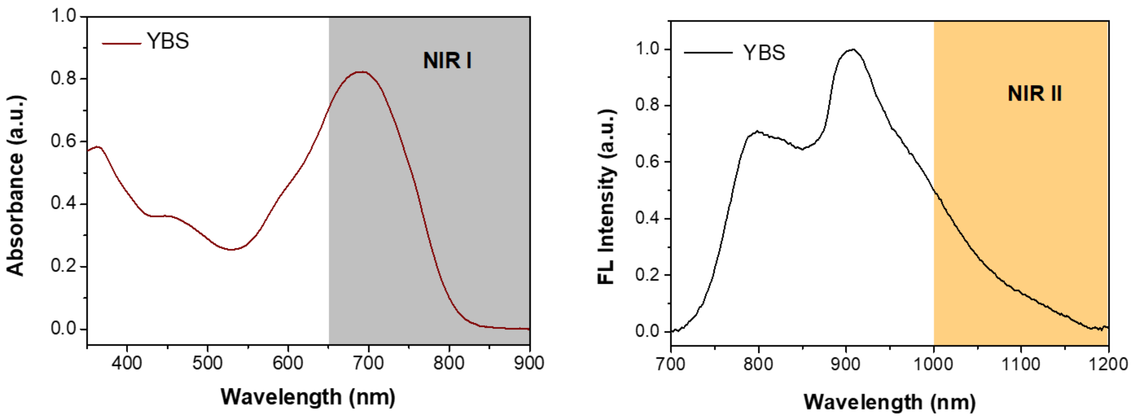

Figure S2. Absorption and fluorescence spectrum of YBS in THF solution.

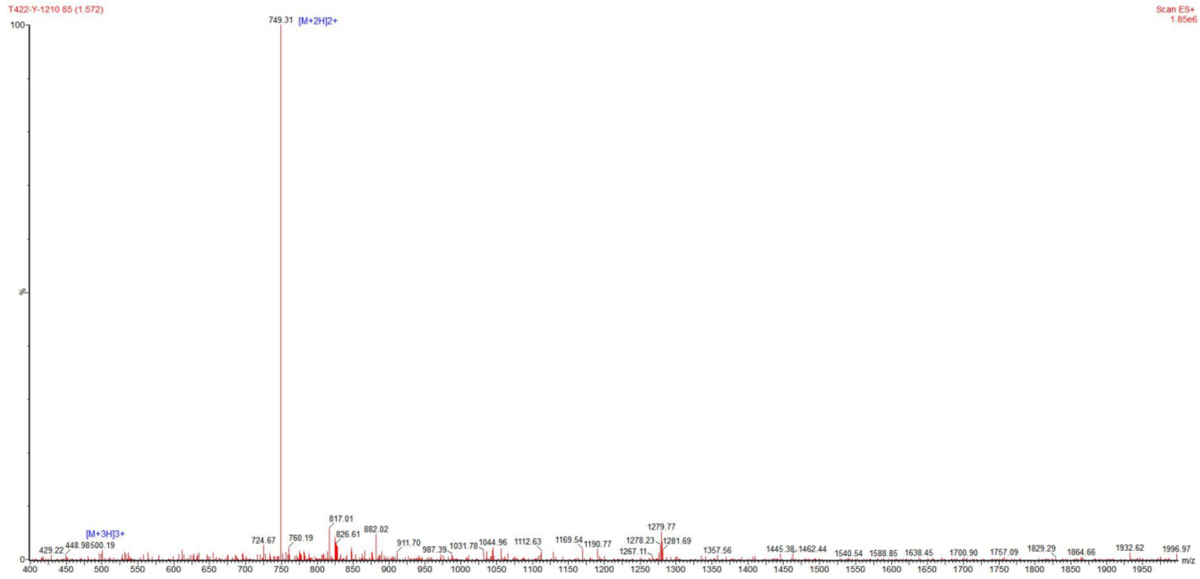

Figure S3. HR-MS spectrum of C-RKRKRKRK-C_{16} peptide.

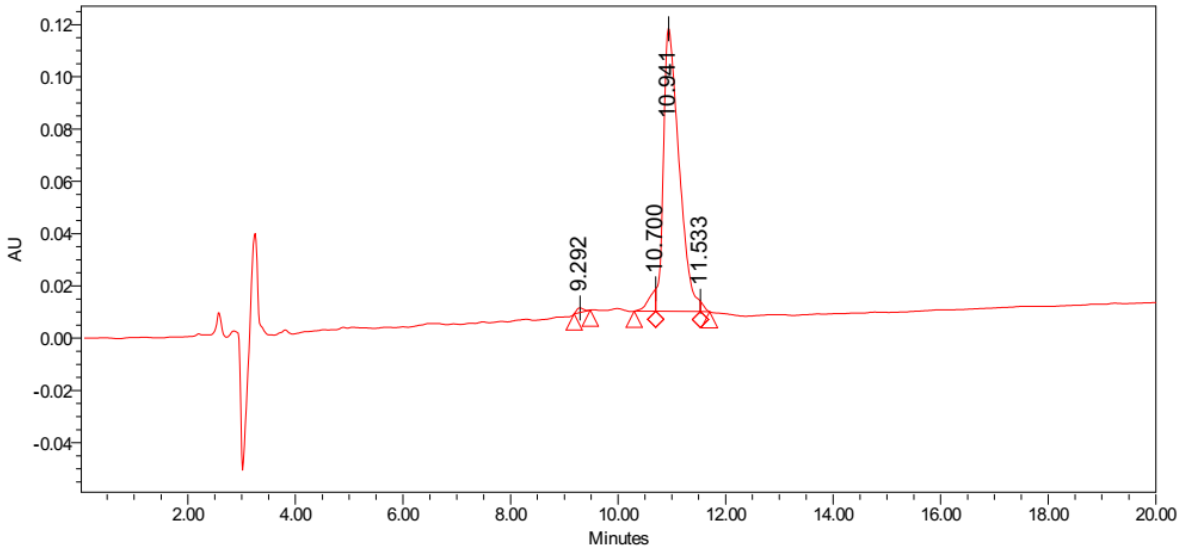

Figure S4. HPLC spectrum of C-RKRKRKRK-C_{16} peptide.

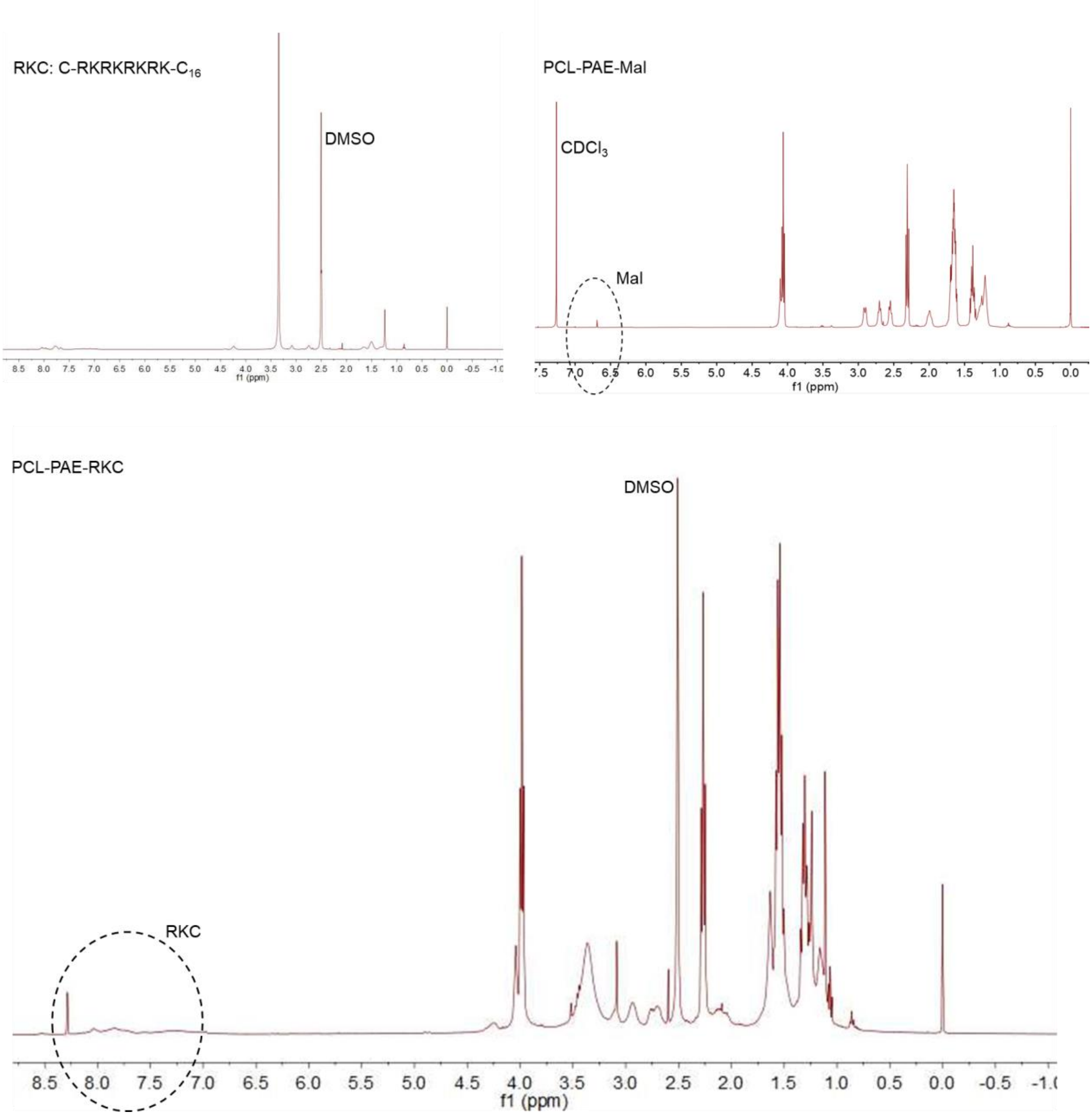

Figure S5. ^{1}H NMR spectrum of RKC peptide in DMSO-d_6, PCL-*b*-PAE-Mal in $CDCl_3$ and PCL-*b*-PAE-RKC in DMSO-d_6.

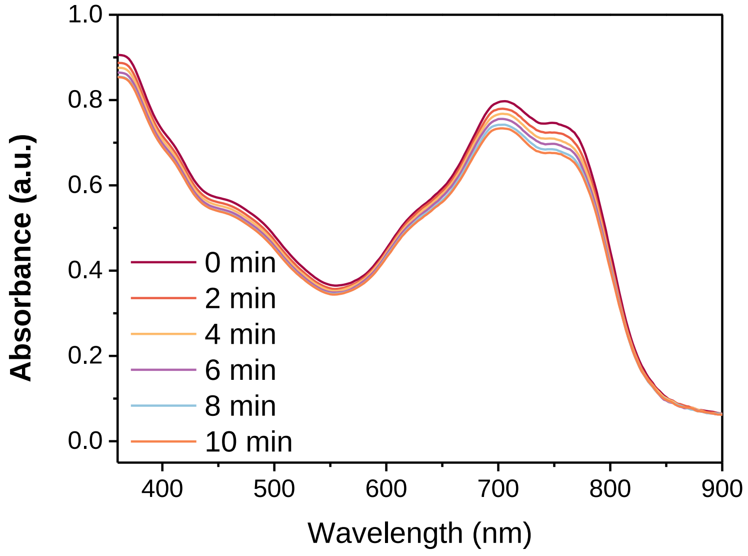

Figure S6. Normalized absorption spectrum of YBS NPs-RKC solutions (YBS: 15 μg/mL) after irradiation with NIR laser for different times.

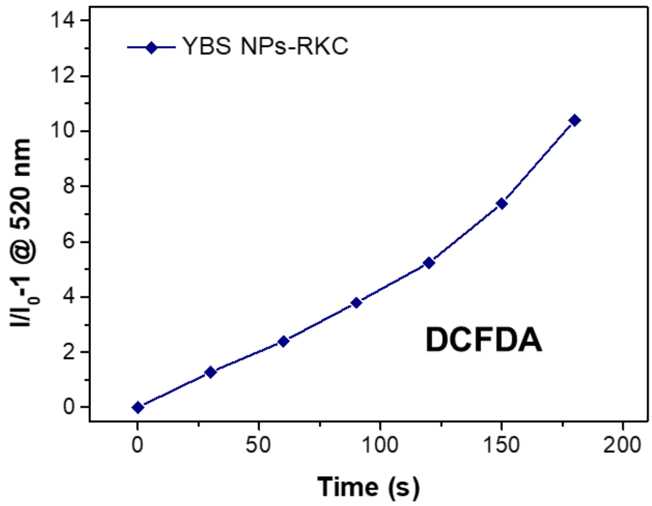

Figure S7. Fluorescence intensity of DCF at 524 nm as a function of light irradiation time of YBS NPs-RKC solutions (YBS: 15 μg/mL).

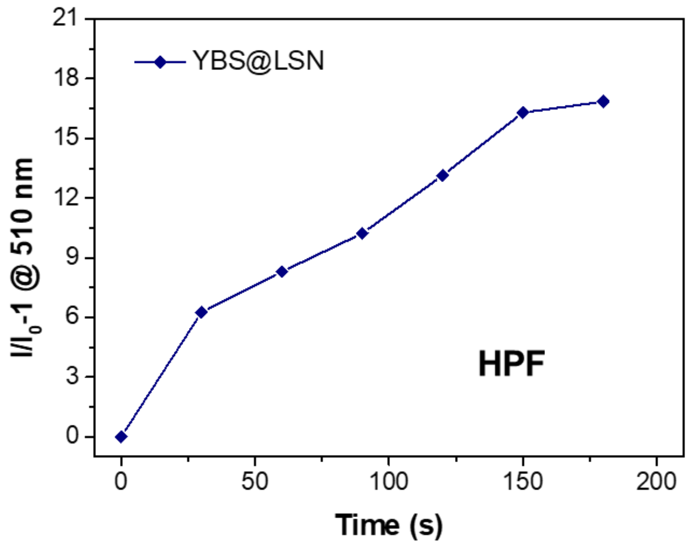

Figure S8. Fluorescence intensity of HPF at 510 nm as a function of light irradiation time of YBS NPs-RKC solutions (YBS: 15 µg/mL).

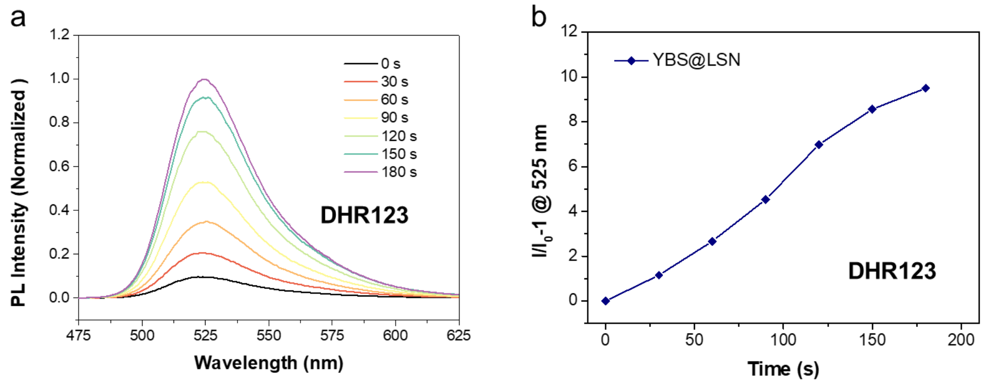

Figure S9. a) Detection of $\bullet O_2^-$ generated by YBS NPs-RKC (15 µg/mL) under NIR laser irradiation (0.3 W/cm^2 at 808 nm) using HPF sensor. b) Fluorescence intensity of DHR123 at 525 nm as a function of light irradiation time of YBS NPs-RKC solutions (YBS: 15 µg/mL).

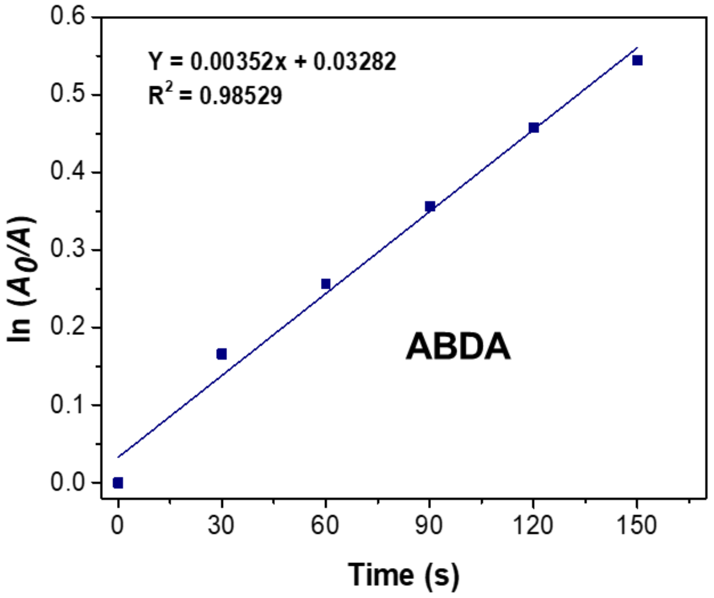

Figure S10. Plot of ln(*A*0/*A*) versus NIR laser irradiation time, where *A*0 and *A* represent the absorbance of ABDA (378 nm) before and after light irradiation, respectively.

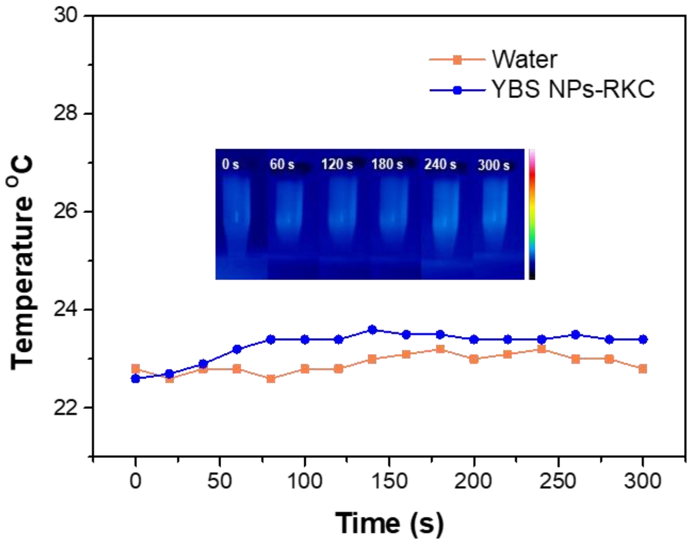

Figure S11. Temperature change of YBS NPs-RKC solutions with increasing light irradiation time.

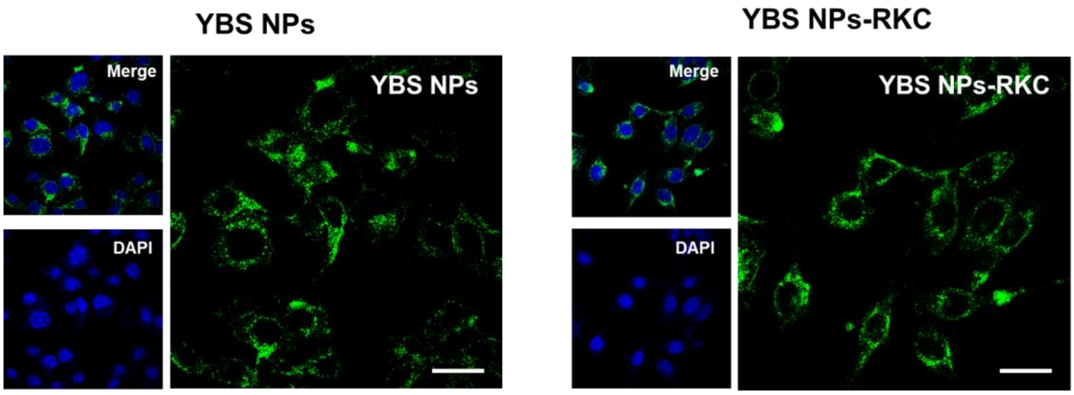

Figure S12. CLSM images of RM-1 cells treated with YBS NPs or YBS NPs-RKC at pH 7.4 at 4 h, respectively. Blue fluorescence: DAPI; green fluorescence: the signals of YBS NPs or YBS NPs-RKC, respectively. Scale bar: 10 μm.

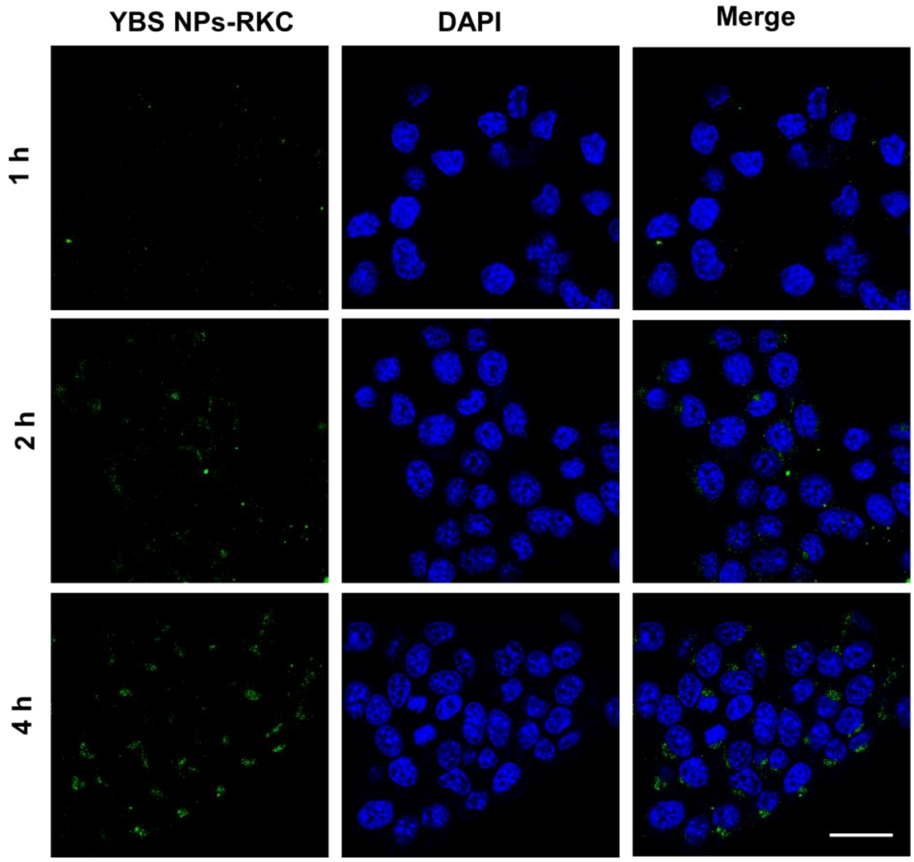

Figure S13. CLSM images of 3T3 cells co-incubated with YBS NPs-RKC at pH 7.4 at different time points, respectively. Blue fluorescence: DAPI; green fluorescence: YBS NPs-RKC, respectively. Scale bar: 10 μm.

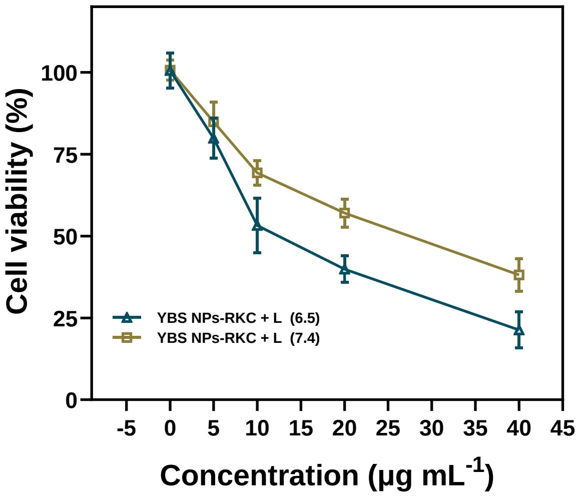

Figure S14. Cytotoxicity of YBS NPs-RKC NPs to RM-1 cells with laser irradiation under hypoxic conditions (2% O_2) .

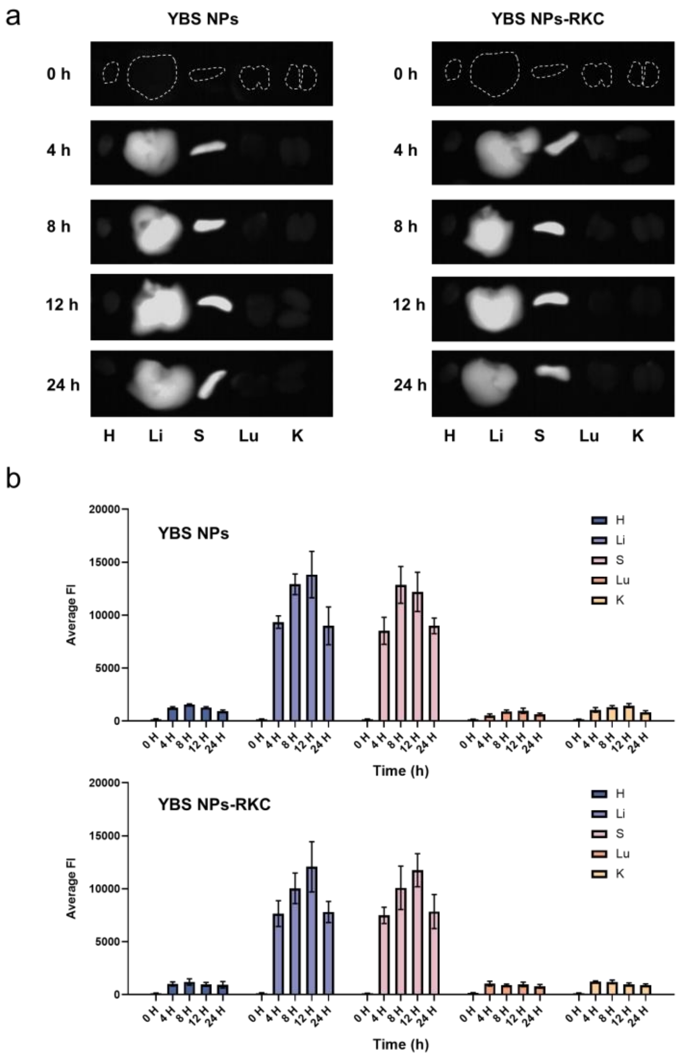

Figure S15. a) Major organ distribution of YBS NPs and YBS NPs-RKC nanoparticles at different time points. b) Quantitative analysis of tissue distribution based on (a) (n = 3).

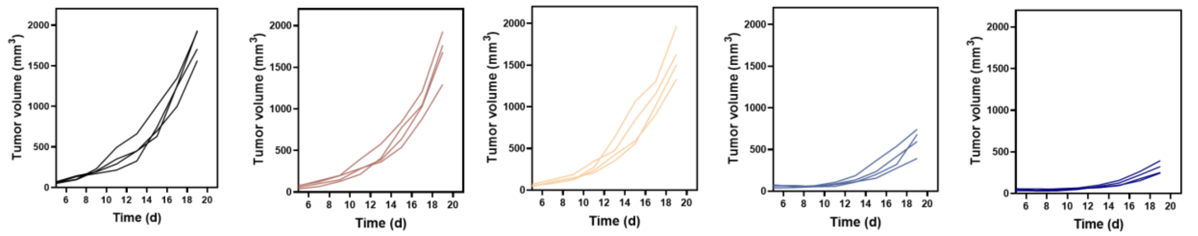

Figure S16. Tumor growth curves of RM-1 tumor-bearing mice under different treatments (n = 4).

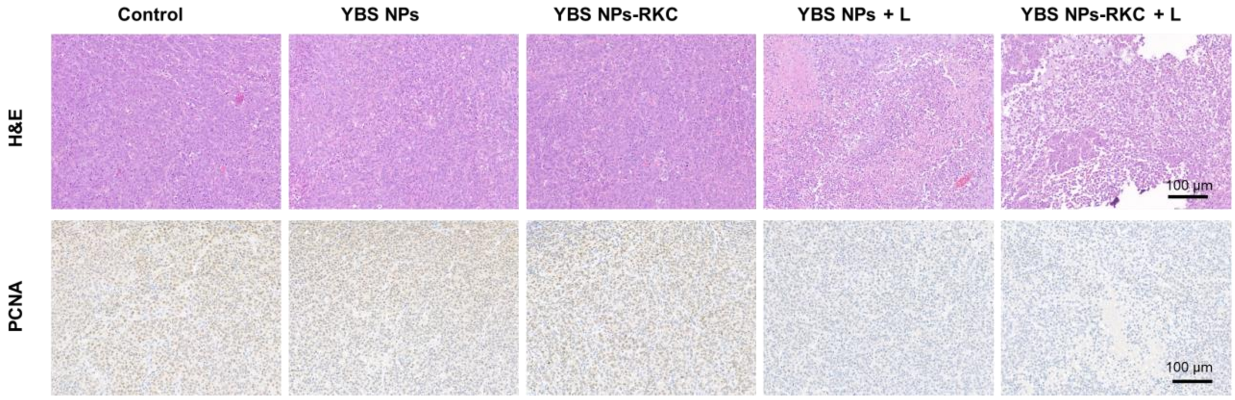

Figure S17. Representative CLSM images of H&E staining and PCNA staining in RM-1 tumor tissues after different treatments. Scale bar: 100 μm.

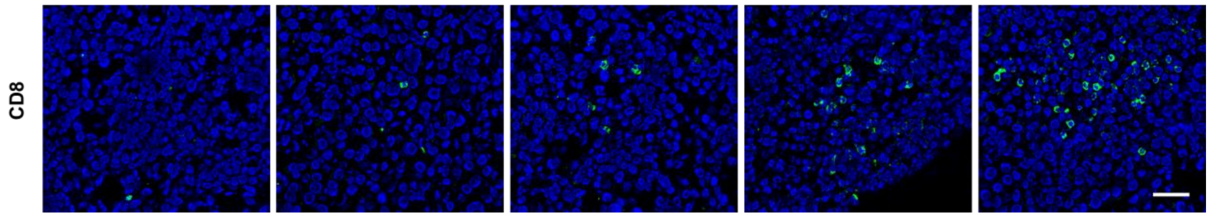

Figure S18. Representative CLSM images of tumor-infiltrating $CD8^+$ T cells in RM-1 tumor tissues after different treatments. Scale bar: 50 μm.

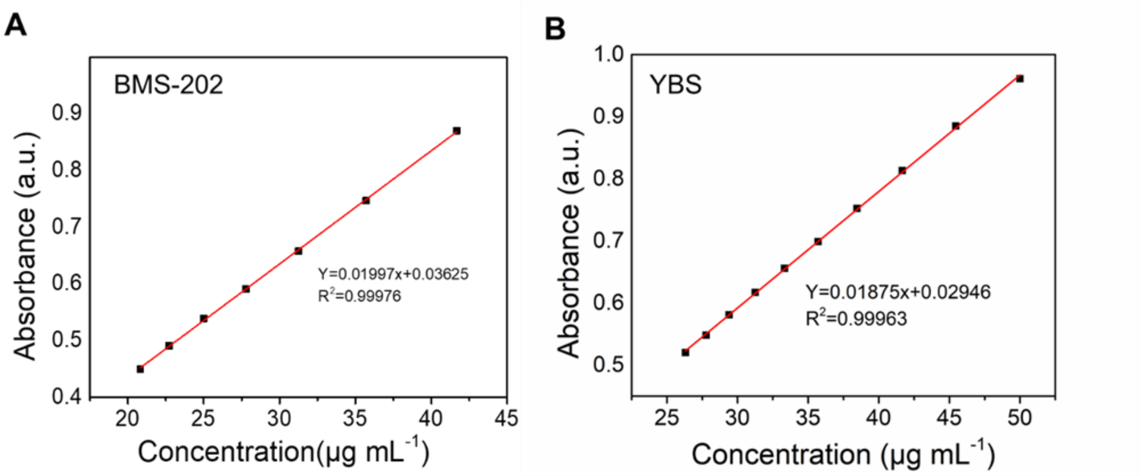

Figure S19. UV/Vis absorption standard curves of (A) BMS-202 and (B) YBS.

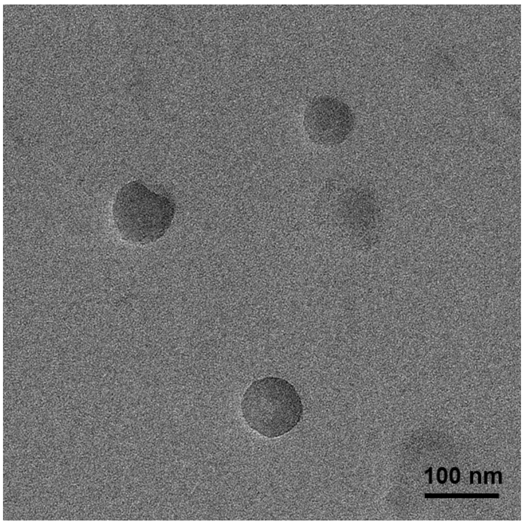

Figure S20. TEM images of YBS-BMS NPs-RKC.

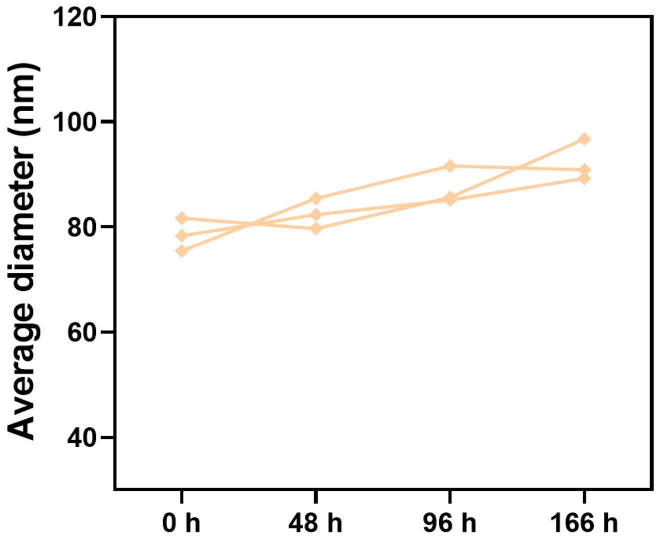

Figure S21. Storage stability study of YBS-BMS NPs-RKC in PBS solution.

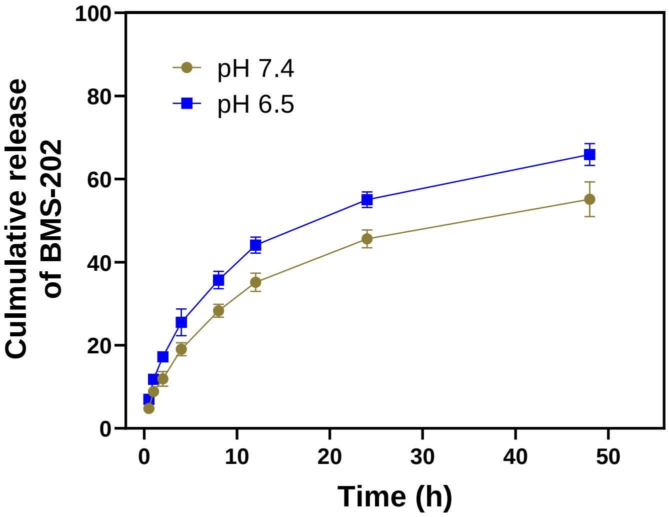

Figure S22. BMS-202 release profiles of YBS-BMS NPs-RKC at different pH values of PBS.

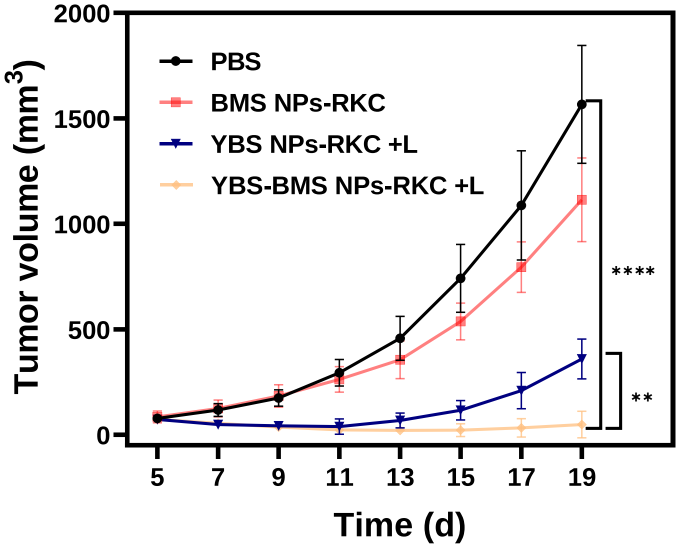

Figure S23. Tumor growth curves in RM-1 tumor-bearing mice model after different treatments (n = 4).

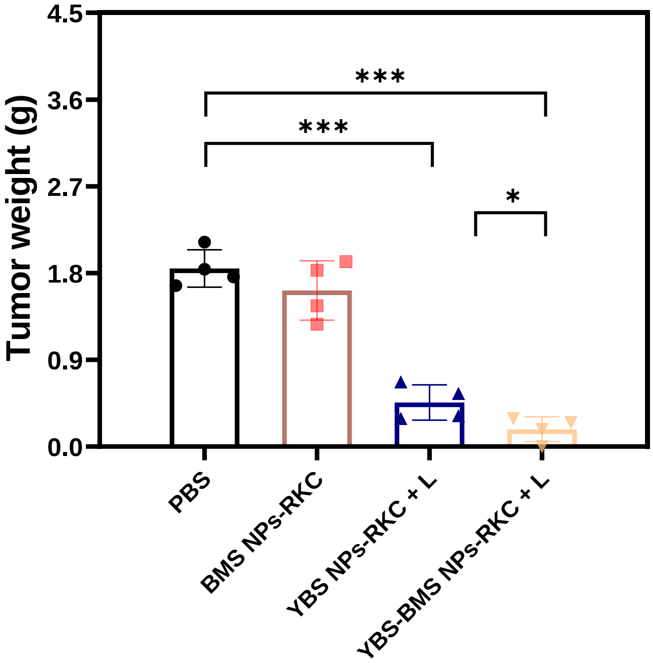

Figure S24. Tumor end weight of subcutaneous RM-1 tumor-bearing mice after different treatments (n = 4).

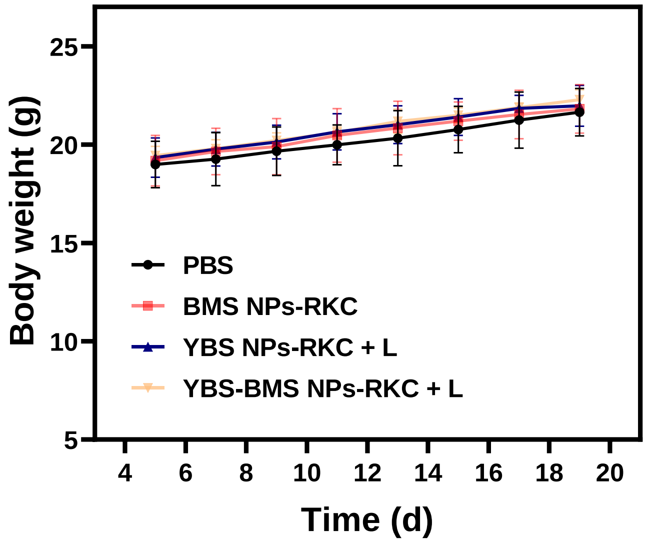

Figure S25. Body weight changes in RM-1 tumor-bearing mice model after different treatments (n = 4).

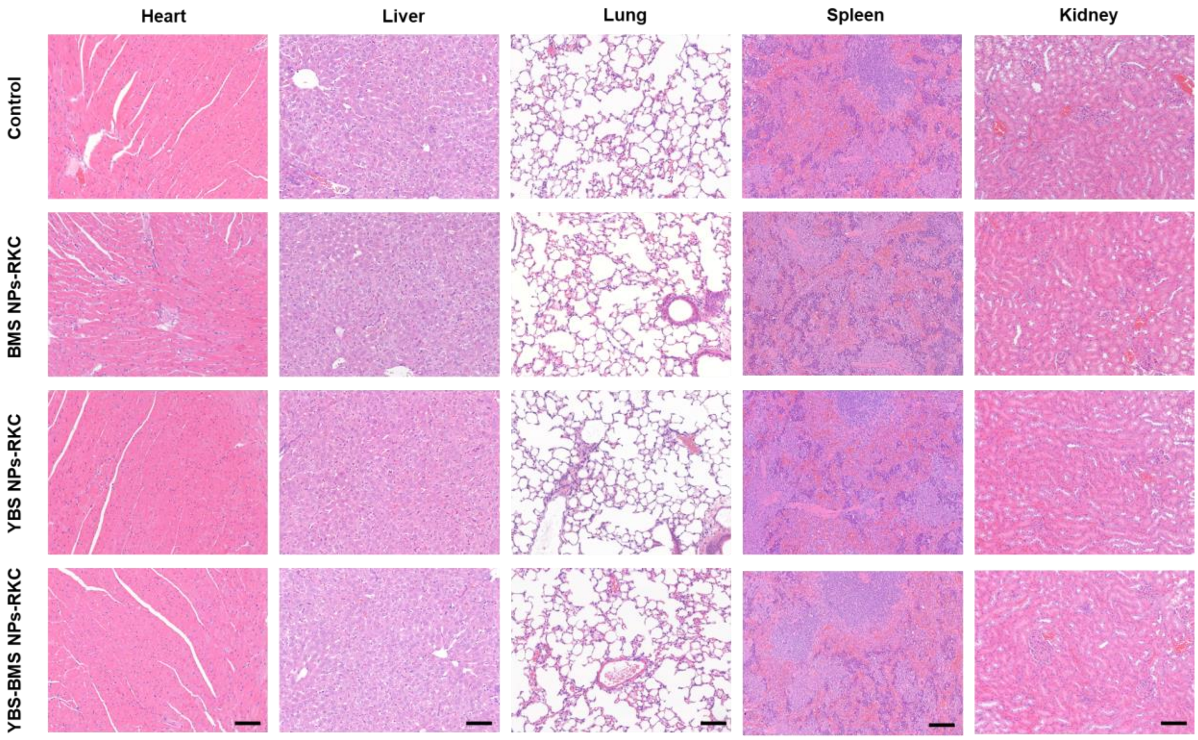

Figure S26. H&E staining of vital organs after different treatments. Scale bar: 100 µm.

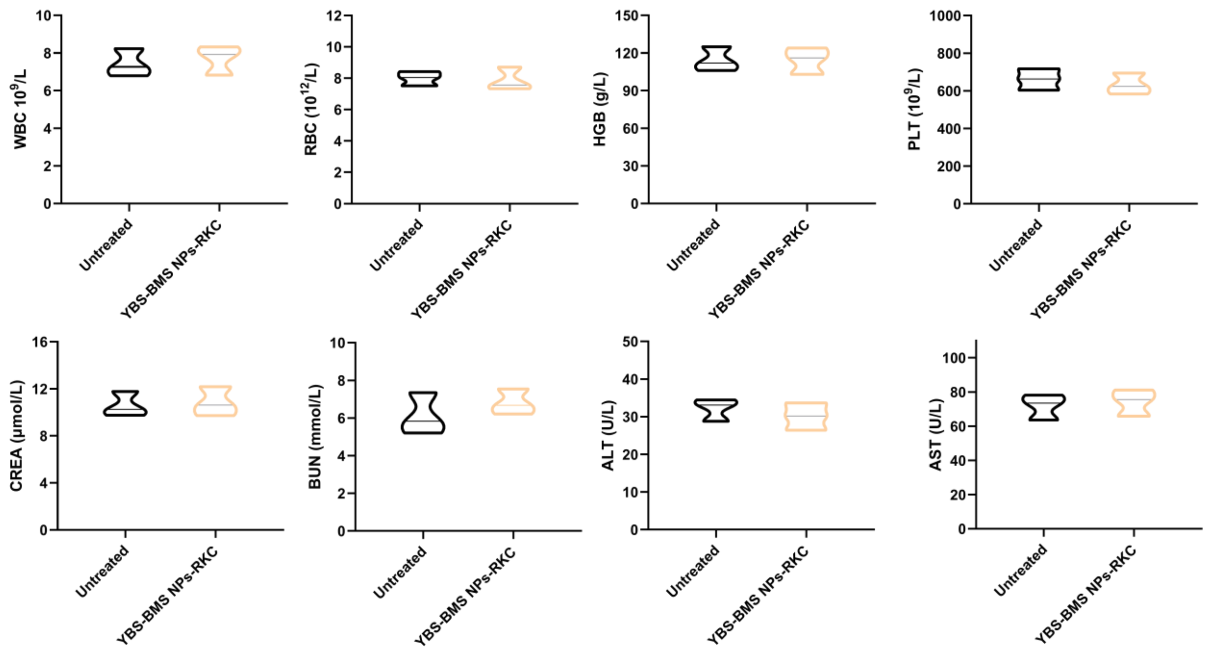

Figure S27. Blood biochemical parameters of the mice with and without intravenous injection of 200 μL of YBS-BMS NPs-RKC (1 mg/mL).

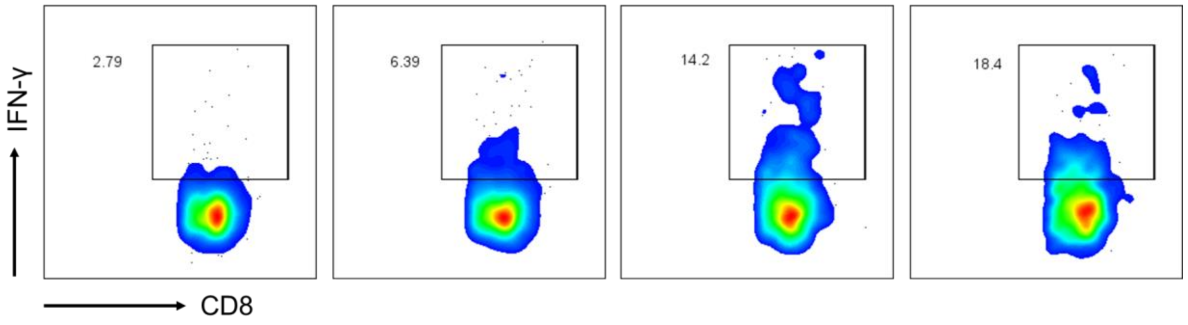

Figure S28. Representative flow cytometry data of IFN-γ^+CD8$^+$ T cells in RM-1 tumor gating on CD3$^+$CD8$^+$ cells.

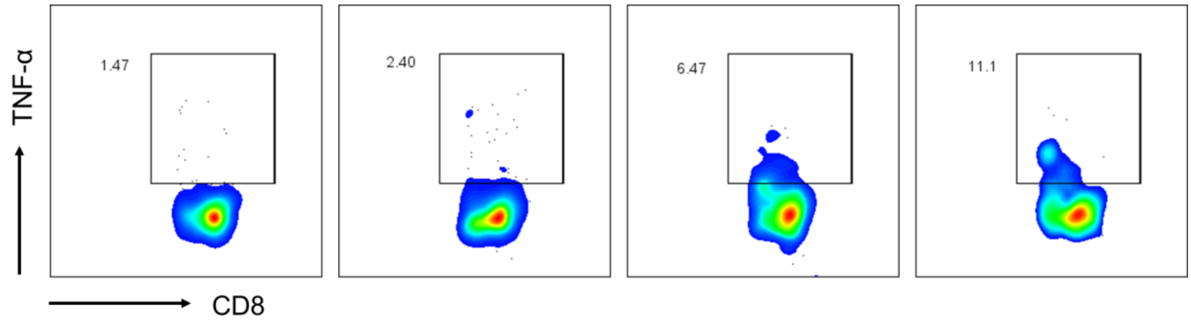

Figure S29. Representative flow cytometry data of TNF-α$^+$CD8$^+$ T cells in RM-1 tumor gating on CD3$^+$CD8$^+$ cells.

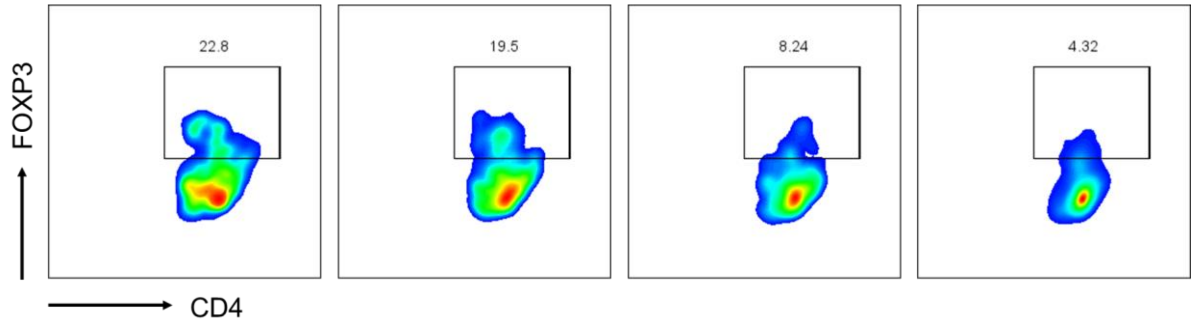

Figure S30. Representative flow cytometry data of Tregs in RM-1 tumor gating on CD3$^+$ CD4$^+$ cells.

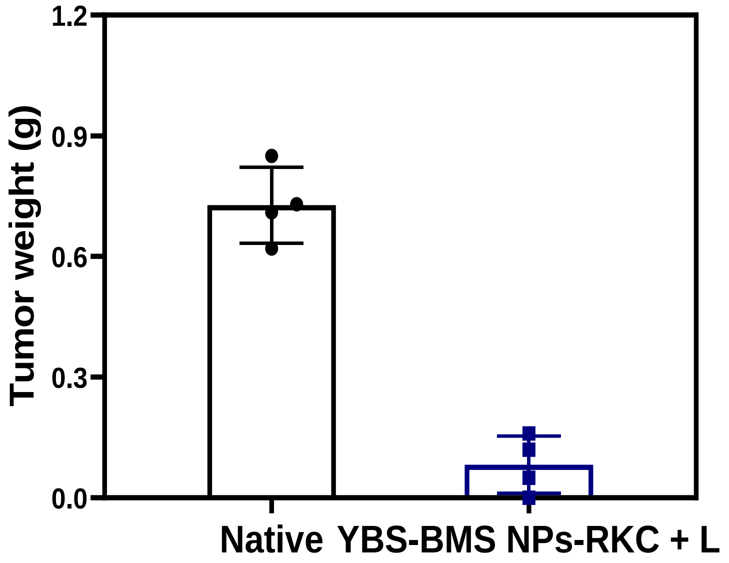

Figure S31. Tumor end weight in PCa re-challenge experiment (n = 4).

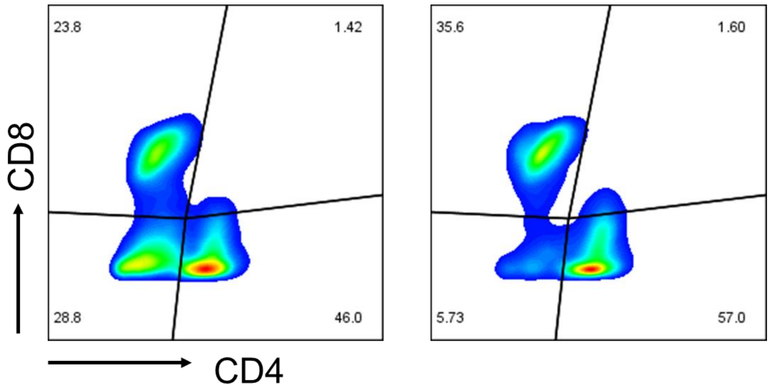

Figure S32. Representative flow cytometry data of $CD8^+$ T cells ($CD3^+CD8^+$) in the spleen of RM-1 tumor-bearing mice at the end of treatment.
